# Brain–Computer Interfaces for Cognitive Rehabilitation: A Narrative Review

**DOI:** 10.3390/bioengineering13101224

**Published:** 2026-10-19

**Authors:** Jessica Cantillo-Negrete, Martín Emiliano Rodríguez-García, Paul Carrillo-Mora, Dalia Danely Méndez-Gómez, Ruben I. Carino-Escobar

**Affiliations:** 1 Technological Research Subdirection, Instituto Nacional de Rehabilitación Luis Guillermo Ibarra Ibarra, Mexico City 14389, Mexico; jcantillo@inr.gob.mx; 2 Division of Research in Clinical Neurosciences, Instituto Nacional de Rehabilitación Luis Guillermo Ibarra Ibarra, Mexico City 14389, Mexico; 3 Bioelectronics Section, Department of Electrical Engineering, Centro de Investigación y de Estudios Avanzados del Instituto Politécnico Nacional, Mexico City 07360, Mexico

**Keywords:** stroke, Alzheimer’s disease, Parkinson’s disease, EEG, dementia, MCI, ADHD, BCI

## Abstract

Cognitive diseases/disorders are a major cause of disability affecting millions of people worldwide due to pathologies or conditions such as Alzheimer’s disease, Parkinson’s disease, vascular cognitive impairment, and attention deficit/hyperactivity disorder. The effects of these diseases/disorders can range from subjective cognitive complaints to mild cognitive impairment and even dementia that can affect multiple cognitive domains, causing disability and alterations in daily functioning and quality of life. Cognitive training has been researched as a potential therapeutic option for treating cognitive disability. In particular, cognitive training delivered using brain–computer interfaces (BCIs) represents a promising technology for cognitive rehabilitation. For this reason, this work presents a narrative review aimed at bridging the clinical aspects of cognition performance and the technical features of BCI systems. Additionally, it reports on the current advances of BCIs for cognitive rehabilitation in the pathologies/conditions that are the main contributors of cognitive impairment globally. Specifically, Alzheimer’s disease, Parkinson’s disease, mild cognitive impairment, cerebrovascular diseases, and attention-deficit/hyperactivity disorder were included in the review; other pathologies that can also cause cognitive impairment such as multiple sclerosis, traumatic brain injury, brain tumors, infectious diseases, and psychiatric conditions such as schizophrenia or autism spectrum disorder, or drug or toxin-related causes were outside the scope of the present review. Across the literature, studies have reported that BCI cognitive training can improve cognition performance for mild cognitive impairment, stroke, and attention-deficit/hyperactivity disorder, showcasing its feasibility as a complementary therapy. However, the amount of evidence is still scarce, with few reported randomized controlled clinical trials and all of the applications comprising non-invasive systems using electroencephalography, while invasive or other non-invasive approaches remain largely unassessed. Therefore, further research regarding the design of BCIs for targeting specific cognitive domains is necessary to allow for the future introduction of these types of systems into clinical rehabilitation environments, rather than only demonstrating their feasibility, as has been mainly done to date.

## 1. Introduction

Cognitive functions can be affected by a range of pathologies involving the central nervous system, leading to cognitive impairment. This condition is characterized by severe difficulty in concentrating, remembering, or making decisions because of a physical, mental, or an emotional condition [1]. The reported prevalence of cognitive-related conditions varies widely across studies and populations, partly due to differences in definitions and assumed methods. For instance, global estimates of cognitive impairment in adults over 50 years of age range from 5.1% to 41% [2]. Other age groups also present a high prevalence of cognitive deficit caused by specific conditions, such as mild cognitive impairment, which has a high estimated global prevalence of 23.7% within the geriatric population [3]. Regardless of this variability, several studies have assessed that there has been an increase in the prevalence of cognitive disability. In the United States, for example, the prevalence of subjective cognitive disability has risen in the last decade from 5.3% in 2013 to 7.4% in 2023, with young adults comprising the age group with the highest increase [1]. Therefore, cognitive disability is a main cause of public health concern worldwide and is aggravated by the absence of a specific treatment strategy, since it will depend on the type of cognitive impairment and its etiology, which are heterogenous. Technology-based cognitive training is a treatment strategy that can be used across a wide range of pathologies causing cognitive impairment [4,5]. This type of training can be provided by means of brain–computer interfaces (BCIs), but it is still a novel, although promising, therapy. Therefore, due to the emergence of BCIs and their application for cognitive training, the present narrative review aims to bridge clinical and technical aspects while synthesizing recent advances and future research directions focused on BCI-based treatment for cognitive impairment. From a clinical perspective, this review aims to assess the scope of diseases or neurological conditions that have been identified as main contributors for cognitive impairment globally. Specifically, this review includes Alzheimer’s disease, Parkinson’s disease, cerebrovascular diseases, MCI, and ADHD, with over 387 million people estimated to present some degree of cognitive impairment due to these diseases/conditions [3,6,7,8]. BCIs applied for cognitive training for these diseases/conditions are also analyzed. Other neurological diseases related to cognitive impairment, such as multiple sclerosis, traumatic brain injury, brain tumors, infectious diseases, psychiatric conditions including schizophrenia or autism spectrum disorder, or drug or toxin-related causes, were outside the scope of the present review. From a technological point of view, the technical specifications needed by BCIs for cognitive training are assessed. This narrative review is organized as follows: First, a background detailing the concepts of cognitive performance, the pathologies affecting it, treatment strategies for cognitive impairment, and BCI technology is presented. Afterwards, the literature search strategy is detailed, along with the synthesized state of the art of BCIs for cognitive training. Lastly, the current literature is critically discussed by identifying the challenges and opportunities of BCI systems for cognitive rehabilitation.

## 2. Background

### 2.1. Cognitive Performance

Cognitive performance refers to the effectiveness of brain functioning and has been characterized into different subdomains. Basic operations, such as sensory operations, are considered as requiring less cognitive resources, compared to other tasks regarded as executive functioning, such as planning or reasoning [9]. A global structure for characterizing cognitive domains was reported by Harvey [9] and is described as follows:

- Sensation is the ability to detect a stimulus within the visual, olfactory, tactile, gustatory, or auditory senses.
- Perception is regarded as the ability to identify previously experienced stimuli using sensory information.
- Motor skills include fine motor abilities like motor speed, reaction time, and dexterity, or global abilities such as balance and equilibrium.
- Construction has been defined as the ability to copy or produce drawings of common objects and involves the conceptualization of visual construction processes. Construction is therefore believed to be related to executive functioning.
- Attention is usually divided into selective and sustained attention; concentration is a type of sustained attention.
- Memory is perhaps one of the largest subdomains of cognitive performance since it encompasses working memory, episodic memory, procedural memory, semantic memory, and prospective memory. Working memory is the ability to consciously hold memory for its adaptive use; episodic memory is the process of encoding, maintaining, and retrieving information about personal experiences or events (which involves a wide variety of types of information, visual, auditory, somatosensory, etc.); procedural memory is the memory related to complex motor activities; semantic memory refers to the process of long-term storage about general knowledge, facts, concepts, or meanings of verbal information; and prospective memory is related to the cognitive ability to remember and perform planned actions in the future.
- The executive functioning subdomain is a group of cognitive processes considered of higher-order because they are responsible for organizing, problem solving, planning, and self-regulating other functions to achieve specific cognitive or behavioral goals.
- Processing speed is defined as the cognitive capacity that defines how quickly the brain assimilates, interprets, and responds to information.
- Language/verbal skills are abilities and processes required to understand and respond to language.

Understanding these cognitive domains is important, since several pathologies can affect the neural processes required to perform the tasks associated with each of the domains.

### 2.2. Pathologies and Conditions Associated with Cognitive Impairment

The alterations in cognitive performance, referred to as cognitive impairment, can hinder the ability to perform activities of daily living [1]. Severe cognitive impairment, manifested by a loss of cognitive skills across several cognitive domains, can be clinically identified as dementia syndrome [10]. It is estimated that worldwide more than 50 million people live with dementia [6]. In particular, around 80% of dementia cases are due to Alzheimer’s disease, which is characterized by progressive cognitive impairment and the presence of β-amyloid-containing deposits in the brain [11]. Although recent immunotherapy treatments have shown potential for reducing cognitive impairment associated with this disease, to date, Alzheimer’s is generally regarded as an incurable disease whose progression might be slowed [12].

After Alzheimer’s disease dementia, Lewy body dementias are the second most common type of dementia [13]. Lewy body dementia is a term that encompasses both Lewy bodies and Parkinson’s disease dementia, since 80% of patients with Parkinson’s disease will also develop dementia [13]. These Lewy body dementias are characterized by the pathological inclusion of α-synuclein within neuronal tissue (Lewy body) and neuronal loss; however, it has been stated that the underlying pathological cause of these conditions is probably multifactorial [13]. Unlike Alzheimer’s disease (AD), where symptom onset is usually a noticeable impairment in the memory domain of cognition, Lewy body dementias most often manifest with the appearance of parkinsonism and cognitive impairment [14].

MCI is a clinical syndrome in which patients’ cognitive performance is significantly deteriorated (1 to 1.5 standard deviations from the expected performance for their age and education) but not at a level that severely affects the capacity to perform activities of daily living. Research related to MCI is important, since it has been estimated that between 23% and 80% of patients with MCI will progress to dementia, most frequently related to an AD diagnosis. Furthermore, the global prevalence of MCI is estimated at 23.7% within the geriatric population [3]. Some patients with Parkinson’s disease dementia, or other types of dementia, have also been observed to first manifest MCI [15,16]. MCI can affect several cognition domains, and it has been identified that certain pathologies are more likely to target specific domains of cognition. For example, in patients with Parkinson’s disease and MCI, the literature reports that the domains of attention, perception, memory, language, and executive functions are more frequently impaired [17]. Fortunately, it has also been reported that MCI can be reversed in 11% to 58% of cases, and that a modifiable factor that can aid in this reversal of the condition is cognitive training [18,19].

Cerebrovascular diseases, ranging from stroke to cerebral small-vessel disease, are recognized as a leading cause of cognitive impairment in the elderly population, with hypertension being one of their primary risk factors [20,21]. The severity of stroke and lesion location, along with the amount of white matter damage, have been associated with the degree of cognitive impairment that patients will present. Cognitive impairments after stroke are presented in most patients, and up to one-third of stroke patients will develop dementia [22]. This is important, since stroke is one of the main causes of disability in the world, including cognitive disability, with millions of patients living with stroke sequelae throughout the world [7]. Something relevant to highlight in stroke is that aphasia affects the language skills of a significant portion of patients that have a left middle cerebral artery stroke, one of the most common blood vessels associated with this condition. Consequently, a large number of stroke patients are likely to have impaired language cognitive skills [22]. Cerebral small vessel disease is a condition that can involve the smallest blood vessels that originate within terminal small arteries, arteriole, arteriovenous anastomosis, capillaries, micro veins, or terminal venules. This disease is usually diagnosed through the discovery of subcortical infarcts, lacunes, white matter hyperintensities, or full regional brain atrophy. Patients with cerebral small vessel disease may be asymptomatic or manifest with MCI or dementia [23].

ADHD is a neurodevelopmental condition characterized by difficulty in maintaining attention and the tendency to be easily distracted (the attention cognitive domain component), excessive talking and a lack of impulse control (the hyperactivity component), or a combination of both [24,25]. This condition is usually diagnosed during childhood, and without proper treatment, it can have a detrimental effect on quality of life across the lifespan. Martin et al. stated that the reported ADHD prevalence is highly variable [26]. Notably, the specific study identified as having a low risk of bias reported the lowest prevalence, which stood at 3.2% in children [8]. The mechanisms suggested to be altered are the mesocortical and mesolimbic cortical circuits, related to attention and reward mechanisms [27]. Genetic and environmental factors have also been hypothesized to contribute to the prevalence of ADHD [27].

A range of other pathologies related to the central nervous system have also been associated with several cognitive alterations, such as traumatic brain injury, amyotrophic lateral sclerosis, or brain tumors, but are outside the focus of this narrative review.

### 2.3. Assessment of Cognitive Performance

Due to the importance of clinically evaluating cognitive performance, several clinical tests have been developed. Examples of widely used tests to screen general cognition are the Mini-Mental State Examination (MMSE) [28], the Montreal Cognitive Assessment (MoCA) [29], or the Mini-Cog test [30]. The MMSE can be administered in 5 to 10 min and is composed of an ordinal scale that has a range of 0 to 30 points [31]. The instrument evaluates several cognitive domains; however, language-related tasks have more importance in the scoring compared to other domains. It has been reported that the MMSE has low sensitivity and high specificity for identifying MCI or dementia, with a cut-off of less than 24 points being suggested for detecting dementia of any severity [32,33]. Although it is widely used across several countries, there have been reports that recommend its phasing-out due to the application of instruments that have more balance in the ponderation of cognitive skills [34]. Originally conceived for the assessment of MCI, the MoCA has a high sensitivity of 0.89 and a specificity of 0.75 for diagnosing this condition [35]. The MoCA is also widely used to screen for dementia, demonstrating a high sensitivity of 0.94 but a low specificity of 0.6 or less [36], although higher specificity has also been reported [37]. The MoCA evaluates the cognitive domains of memory, language, attention, orientation, and executive function, generally requiring more than ten minutes for its application. It has an ordinal scale from 0 to 30 points, where lower scores indicate greater cognitive impairment, with a cut-off value below 23 points recommended for identifying MCI [14]. The Mini-Cog test is composed of a delayed three-word recall task and a clock drawing test. It can be applied in a very short time, taking between 3 and 5 min to complete, and has the main advantage of having little bias due to education or language [30]. Although less reported for cognitive impairment diagnosis compared to the MMSE and the MoCA, it has been reported that the Mini-Cog test can have a sensitivity of 73% and a specificity of 84% within the primary care setting [38]. In addition, recent cognitive tests have been designed for their specific application using digital devices. These digital cognitive assessments have been proposed as a significant advancement in diagnosing cognitive impairment. By increasing objectivity and allowing for remote administration, they reduce the risks and costs associated with travelling to primary care facilities for the assessment of cognitive impairment [39].

### 2.4. Treatment Strategies for Cognitive Impairment

Treatment strategies have been proposed for cognitive impairment and can range from pharmacological to lifestyle changes. For example, for the case of AD cognitive impairment, acetylcholinesterase inhibitors, memantine, some anti-diabetic drugs, and, recently, monoclonal antibodies have been used, but there is still insufficient evidence to state that these interventions can slow the progression of cognitive impairment [40]. Other complementary interventions have been proposed for AD cognitive impairment such as physical exercise, cognitive training, dietary modifications, aromatherapy, art therapy, and electrical stimulation, but further evidence is required to fully assess their efficacy [40]. For the case of cognitive impairment related to cerebrovascular diseases, most of the reported interventions have been pharmacological, while a smaller number consist of physical devices and rehabilitation strategies [41]. These include transcranial magnetic stimulation, acupuncture, light therapy, physiotherapy, cognitive rehabilitation, and computerized cognitive rehabilitation [41]. Some of the most used complementary strategies for the treatment of cognitive impairment can be classified as cognitive training, which is defined as interventions that use cognitive tasks or intellectually demanding activities, with its main goal being enhancing general cognitive ability [42]. Computers have allowed for the introduction and application of computerized cognitive training, which has also been researched as a complementary form of cognitive impairment treatment [43]. Furthermore, the advancement of computer and bioinstrumentation technologies have allowed for the development of systems that provide real-time feedback of physiological information to users, usually through a computer monitor [44]. These types of systems are known as biofeedback systems, or neurofeedback if the information is extracted from the nervous system [44]. The application of neurofeedback for cognitive rehabilitation has been reported across a range of pathologies/conditions [45,46,47,48]. More recently, BCI-based interventions have emerged as a promising complementary therapy for the treatment of cognitive impairment. In Table 1, current approved treatment strategies for several types of cognitive impairment are summarized [49,50,51,52].

### 2.5. Brain–Computer Interfaces

The BCI society has recently defined a BCI as follows: “A system that measures brain activity and converts it in (nearly) real-time into functionally useful outputs to replace, restore, enhance, supplement, and/or improve the natural outputs of the brain, thereby changing the ongoing interactions between the brain and its external or internal environments. It may additionally modify brain activity using targeted delivery of stimuli to create functionally useful inputs to the brain” [53]. Despite this recent consensus, the historical development of these systems began much earlier. Graimann et al. identified that the earliest BCI-like system was pioneered by Dr. Grey Walter in 1964. In that study, a patient who underwent a surgical procedure with electrodes implanted in the motor cortex successfully controlled the change of slides presented in a screen using neural information acquired from those electrodes [54]. BCIs are composed of four different components: signal acquisition, feature extraction, feature translation, and device output commands that can also provide feedback, in addition to a strategy or paradigm for encoding users’ intentions and translating them afterwards into commands [55]. These components are described in the following paragraphs.

Signal acquisition can be performed by inserting measurement devices directly into brain tissue, a modality known as invasive BCIs. Alternatively, non-invasive BCIs acquire brain information from devices that are placed over the scalp or sensors that do not even require direct contact with the body of the user. Invasive BCIs can perform signal acquisition using either intracortical recordings, which use needle-shaped electrodes that are allocated within the cortex, electrocorticography (ECOG), which uses electrodes fixed to a flexible housing that are placed directly over the cortex [54], or within blood vessels using vascular stent electrodes that can also provide deep brain stimulation [56]. On the other hand, non-invasive BCIs are mostly composed of electroencephalography (EEG) recordings, which measure the added electrical activity of millions of neurons recorded from electrodes that are placed over the scalp. EEG electrodes have evolved to become more technologically sophisticated, with active electrodes that have impedance coupling and preamplification circuits built within them, now common for research applications [54]. Other less common non-invasive techniques for BCIs are magnetoencephalography (MEG), functional near-infrared spectroscopy (fNIRS), and functional magnetic resonance imaging (fMRI). MEG involves the measurement of small magnetic fields produced by neurons’ electrical activity [57]. fNIRS is based on the deflection and absorption of near-infrared light within the brain tissue from which oxyhemoglobin and deoxyhemoglobin concentrations can be computed [58]. fMRI uses a strong magnetic field to measure blood oxygenation and flow using the blood oxygen-level-dependent signal [59]. Figure 1 illustrates invasive and non-invasive signal acquisition technologies used in BCIs.

After signal acquisition, features relevant to the user’s intentions need to be extracted. The paradigms used to elicit brain activity related to these intentions can range from evoked potentials to the motor imagery of a limb. Two of the most widely used evoked potentials paradigms are P300 and Steady-State Visual Evoked Potentials (SSVEPs). P300 is a positive deflection seen in the EEG, mainly in the parietal and central positions of the 10–20 system, after approximately 300 ms of the presentation of an uncommon or target stimulus and is known as oddball [60]. SSVEPs are elicited when a visual stimulus, at a frequency usually above 8–10 Hz, is presented to the BCI user [61]. These stimuli elicit frequency components observed in the EEG in the same frequencies as the stimuli, and users, for example, can select with their gaze a visual stimulus within a computer screen [61].

Other widely used paradigms are those based on sensorimotor rhythms (SMRs), which can be elicited by users through movement, passive movement, for example by a robotic orthosis, or with the motor imagery of a limb [62]. These paradigms usually involve the decoding of motor-related information from the central–parietal channels of the 10–20 system with patterns showing across alpha and beta rhythms and the mu rhythm, a special motor-related oscillation seen within the 8–12 Hz range. Although the limited spatial resolution of the EEG limits the number of commands that can be elicited through SMRs, it is feasible for users to control a BCI with two to four commands using this paradigm [63]. Event-related desynchronization/synchronization are features computed from the relativized frequency power during movement-related tasks, such as motor imagery or motor intention, and are widely used for controlling external devices in BCI systems [64]. These control paradigms, as well as the stages of a BCI, are depicted in Figure 2.

Once users’ intentions are encoded in the acquired brain signals, computational algorithms will be needed to separate the information related to these intentions from other sources of information that can be found within an EEG recording, such as muscle artifacts or brain activity unrelated to users’ intentions. Temporal and spatial filters can be applied to the signal for preprocessing to eliminate this noise. Subsequently, feature extraction is performed to obtain relevant neural characteristics. Depending on the BCI paradigm, this includes extracting time-domain components like P300, spatial features via the common spatial patterns (CSPs) algorithm, frequency-domain power for SSVEPs, or alpha and beta power computed with wavelet transform for motor-related activity [55]. The feature translation converts the extracted features into command outputs. This stage requires machine learning algorithms to translate features in the high-dimensional space of channels–time–frequency into two or more command outputs [65,66]. After feature translation, the last stage of a BCI is composed by the transmission of the obtained command outputs to an external device that can range from a computer cursor to a robotic assistive device. Wireless transmission of these command outputs is now widely reported in several BCI applications, even in invasive BCIs [66,67]. These output commands provide feedback to the patient, such as the onset of a stimulation agent or even the activation of a device aimed at neurorehabilitation such as a robotic orthosis [55].

At this point, it is important to highlight the distinction between a BCI system, a conventional neurofeedback system and an open-loop system for neuromodulation. Despite being commonly confused in the literature, they represent distinct modalities of neurotechnology, and the scope of this review focuses strictly on BCI applications.

A BCI requires the execution of tasks that allow the user to control an external device. Biofeedback has been defined as a process that provides real-time information from psychophysiological recordings about the levels at which physiological systems are functioning [44]. Therefore, biofeedback, specifically neurofeedback, is related to monitoring brain activity and showing this activity in real time to the user [44]. Open-loop neuromodulation provides neurostimulation with fixed and preprogramed parameters that do not depend on any neurophysiological information that is acquired from the patient during the intervention [68]. BCI systems have to decode users’ intentions from brain activity in order to control an external device; furthermore, a BCI should encompass all the stages described in Figure 2, including the classification of different tasks performed by the user based on features extracted from neurophysiological data [44]. Open-loop neuromodulation will depend solely on the prefixed parameters.

An example of a BCI would be a system that acquires EEG recordings during a hand motor imagery task in 1 s windows, then extracts frequency-related features such as frequency power in alpha, and classifies between hand motor imagery, or not, in each of the windows by inputting the features into a pretrained classification model; if motor imagery is classified for a window, a robotic hand orthosis attached to the patient’s hand is actioned, providing passive finger movement. An example of a neurofeedback application would be a system that acquires EEG recordings, computes the power in the alpha band of a 1 s window, and transmits the value of this power continuously to a computer monitor that displays a bar that becomes taller or shorter depending directly on the magnitude of the extracted alpha power. An open-loop neuromodulation system could consist of applying neurostimulation, such as an injected electrical current or a magnetic field, based on a preprogrammed routine, without the patient having to perform any action.

## 3. Search Strategy

In accordance with the SANRA guidelines for narrative reviews, research articles in peer-reviewed journals published up to June 2026 were independently assessed by two researchers [69]. After study identification, the selected papers were collated by both researchers. Any disagreements regarding study eligibility were resolved through discussion or by consulting a third researcher. A stratified and iterative search strategy was conducted across multiple databases to ensure a comprehensive coverage. Lists of articles’ titles and abstracts were exported as .csv files and text files, respectively. The consulted databases, search syntax and specific dates of search are shown in Table 2.

Inclusion criteria were research articles, clinical trials, case series and case studies, written in any language, where a BCI was deployed for cognitive rehabilitation in any of the assessed pathologies/conditions. In cases of manuscripts written in languages other than English, generative artificial intelligence models were used for translation. Exclusion criteria were protocol descriptions, review articles, and studies that employed a neurofeedback system, or that lacked information to assess the methodology that was employed for cognitive rehabilitation. In order for an intervention to be classified as a BCI intervention, the four stages that compose BCI systems needed to be described, including the classification between tasks. Conversely, borderline applications, such as a system that provided real-time feedback derived from an extracted feature level rather than directly mapping its magnitude to the feedback input, were included for analysis.

The initial database search yielded 6582 studies, with 4033 duplicates. Duplicates were removed after their identification within a .csv file that encompassed exported titles for each database. After removing duplicates, the remaining 2549 were screened; based on title and abstract information, 67 articles seemed to meet criteria and underwent full-text review. After full-text revision, 17 articles strictly met the inclusion and exclusion criteria. From the eliminated studies at this stage, 10 were eliminated due to employing neurofeedback systems instead of a BCI for cognitive neurorehabilitation. Figure 3 illustrates the literature search and selection.

## 4. Cognitive Training with Brain–Computer Interfaces

### 4.1. Current Evidence Across Pathologies or Conditions

Features of the revised studies are summarized in Table 3. Of the reviewed pathologies or conditions, BCI-based cognitive training for AD was the one for which the least evidence has been reported. Although some studies have reported clinical trials or case series involving AD dementia using neurofeedback systems that resemble a BCI, no peer-reviewed articles reporting the application of a BCI system for AD cognitive rehabilitation were identified within the literature [45,70,71]. For Parkinson’s disease, the perspective was similar to AD, with only one study reporting the use of a BCI for cognitive rehabilitation in only three patients [72]. The BCI system used in this study was classified as borderline with a neurofeedback application, since the patients’ level of event-related desynchronization was the metric used to provide different types of visual feedback from a first-person perspective. The lack of studies targeting AD or Parkinson’s is likely related to the difficulty for patients with dementia and motor impairment to control a BCI system.

Conversely, stroke was the pathology with the largest number of identified studies; a total of 203 stroke patients were reported to have received BCI-based cognitive training across studies utilizing controlled designs. This number of patients was computed by taking into account all patients who received a BCI intervention, including those who received a BCI intervention plus other therapies, even if the BCI intervention was part of the control group. Patients who received a sham BCI intervention were excluded because they did not control their BCI feedback. Among studies without a control group, three focused specifically on stroke-related aphasia, reporting recovery of the targeted language/verbal skills and reductions in aphasia symptoms, but involved small sample sizes, totaling 22 patients [73,74,75]. Three out of six of the controlled studies used the MoCA for cognitive performance assessment, reporting average MoCA score increases as high as two points [76,77,78]. An increase of 1 to 2 points in the MoCA has been stated as a minimal clinically important difference in stroke population [79]. Therefore, the evidence suggests that stroke patients that engage in BCI-based cognitive rehabilitation may have improvements in their cognition domains, with perception, construction, and memory cognition domains being stated as improving in two studies [78,80]. Only two studies compared a BCI-based intervention with conventional cognitive therapy, with a higher cognitive performance reported with the BCI-based intervention in both of them [76,80]. Furthermore, some of the controlled studies involving stroke combined a BCI with other types of cognitive interventions, such as conventional therapy or biofeedback, limiting the number of studies that have assessed the cognitive effects of solely BCI-based therapies [78,81,82]. This implies that evidence that supports the inclusion of a BCI as a complementary therapy for stroke cognitive rehabilitation is still scarce compared to evidence on the use of a BCI for motor rehabilitation, which includes at least 886 patients within 21 randomized controlled trials [83]. Other cerebrovascular diseases have not been targeted for cognitive rehabilitation, implying that the effects of BCI-based cognitive training have not been reported in complete populations of cerebrovascular disease patients, such as those with small-vessel disease, with this being another cerebrovascular disease for which BCI-based cognitive interventions could have the most potential due to these patients usually presenting MCI.

Only two studies for BCI-based cognitive training were identified for MCI or elderly populations, with only the study involving elderly populations utilizing a controlled design. Thus, following the same computation as for stroke patients as previously described, only 63 patients have received BCI interventions to date, with significant recovery reported mainly in the perception, language/verbal skills, memory, and executive functioning domains [84,85]. The lack of more studies targeting this population is hard to understand, since populations with neurological pathologies that involve more complex disability scenarios, such as stroke patients, with mild to moderate cognitive impairment have effectively used BCI systems.

Some evidence has been reported regarding the positive cognitive effects of BCIs for ADHD, driven mainly by Cuntai Guan et al. To date, a total of 296 ADHD patients have been engaged in BCI-based interventions, applying the same calculation criteria used for stroke and MCI patients that have engaged in BCI interventions [86,87,88]. As could be expected, attention was the targeted cognition domain within these therapies and showed significant improvement across the studies. Therefore, as with stroke, evidence for ADHD seems to be stronger compared to the other assessed pathologies or conditions of this review but is still derived from a limited sample of studies.

### 4.2. Assessment of Cognitive Rehabilitation

High heterogeneity was observed in the outcome measurements reported within the assessed studies. Although some assessments, such as the ADHD-Rating Scale, are condition-specific, the inclusion of a generalized secondary cognitive assessment could allow us to directly compare the clinical effects of BCI-based intervention studies across pathologies and BCI technical designs. The MoCA could serve such purpose, since it has already been used to assess cognitive performance in several BCI studies targeting stroke [76,77,78]. Furthermore, the MoCA has reported thresholds for minimal clinically important differences, as well as for the diagnosis and identification of dementia or MCI, while evaluating a wide range of cognitive domains [29,79]. However, the MoCA cannot replace condition-specific and domain-specific assessments, such as those required for ADHD studies. Furthermore, it is important to highlight that, to date, no studies have employed other cognitive screening tools such as the ACE-III scale [89,90]. This scale offers the significant advantage of a broader scoring range (100 points) compared to the MoCA, while also allowing for independent assessment and analysis across five cognitive domains (attention/orientation, memory, verbal fluency, language, and visuospatial function); consequently, it may be more sensitive to changes resulting from BCI interventions. Although it has seen limited use in BCI research, likely because it takes longer to administer and requires more extensive training for both administration and interpretation [89], it remains an instrument worth considering for future studies.

**Table 3:** Studies that fulfilled selection criteria and reported the effects of BCI-based cognitive training in pathologies/conditions associated with cognitive impairment. The main outcome measures related to cognitive performance assessment are summarized for each study. Mini-Mental State Examination (MMSE). Test battery for Attention Performance (TAP). Aachen Aphasia Test (AAT). Montreal Cognitive Assessment (MoCA). Loewenstein Occupational Therapy Cognitive Assessment (LOTCA). Attention Network Test (ANT). Attention Deficit/Hyperactivity Disorder (ADHD). Conners Continuous Performance Test–Third Edition (CPT-3).

Study	Experimental Design/ Follow-Up	Experimental Group (*n*, Age)	Control Group (*n*, Age)	Condition	Feedback Type	Cognitive Assessment Instruments	Cognitive Domains That Presented Changes
Turconi et al. [72]	Uncontrolled. No follow-up	*n* = 3, age range: 71–77 years	No	Parkinson’s Disease	Visual feedback with an avatar with walking speed increasing using imagined walking	MMSE (cognitive screening); Interference task of the Stroop’s Test; Part B of the Trail Making test (executive functions) and phonemic fluency task (executive control and language)	Improvements in attention and executive functioning. MMSE scores increased by one point in a patient and by two points in another. They remained unchanged for the other patient. Stroop test and phonetic fluency increased in all three patients.
Gomez-Pilar et al. [84]	Controlled, randomized after accounting for age and gender, blinding not reported. No follow-up	*n* = 31, mean age = 68.3 ± 4.3 years	*n* = 32, mean age = 68.0 ± 5.6 years Did not receive the BCI-based intervention	Old age > 65 years	Visual feedback with 5 different types of tasks that involved the selection of two outputs using right and left-hand motor imagery	Luria-AND test includes nine subtests of five different cognitive domains: visuospatial (visual perception and spatial orientation), oral language (receptive and expressive speech), memory (immediate memory and logical memory), intellectual (thematic draws and conceptual activity), and attention (attentional control)	Improvements in perception, language/verbal skills, memory and executive functioning. Significant higher Luria-AND scores after the intervention for the BCI intervention group within the visuospatial, oral language, memory and intellectual functions compared to the control group.
Mendoza-Laiz [85]	Uncontrolled. No follow-up	*n* = 32, mean age = 68.2 ± 4.3 22 were in the 61–69 years age group and 10 were in the 70–81 years age group	No	Mild cognitive impairment	Visual feedback with 5 different types of tasks that involved the selection of two outputs using right and left-hand motor imagery	Luria-AND test includes nine subtests of five different cognitive domains: visuospatial, oral language, memory, intellectual and attention (as previously described)	Improvements in perception, construction, language/verbal skills, memory and executive functioning skills. Significant higher Luria-AND scores after the BCI intervention were observed within the younger group in almost all assessed functions, except for attention and logical memory.
Kleih et al. [74]	Uncontrolled. No follow-up	*n* = 5, mean age = 58.6 ± 14.5	No	Stroke	Visual feedback using a BCI speller with P300 as the paradigm	German Attention Performance Test (TAP) Software, package 2.3. Subtest: Alertness, Divided attention, Selective attention and Flexibility.	Changes within domains were not qualitatively assessed. At least a 60% spelling success rate could be achieved with the BCI by patients.
Tonin et al. [91]	Uncontrolled. No follow-up	*n* = 3, age range: 46 to 61 years, median = 57 years	No		Visual feedback with the selection of two possible outputs related to a manual task employing scissors	Italian clinical neuropsychological test for the assessment of visuospatial exploration in peripersonal space by a Symbol cancelation task	Improvements in perception and construction. Reaction time of a button pressing task decreased after the intervention.
Kleih et al. [73]	Uncontrolled. Follow-up at three months	*n* = 7, mean age = 57.4 ± 11.2	No		Visual feedback using a BCI speller with P300 as the paradigm	German Attention Performance Test (TAP) Software. Subtest: Alertness, Selective Attention and Sustained Attention. Aachen Aphasia Test (AAT) six subtests: Spontaneous Speech, Token Test, Repetition, Written Language, Denomination, and Speech Comprehension	Improvements in language/verbal skills, and reduced aphasia symptoms. TAP test showed no significant changes in attention. AAT scores increased in most of the analyzed patients.
Musso et al. [75]	Uncontrolled. Follow-up at three months	*n* = 10, mean age = 58.4 ± 12.1 years	No		Auditory feedback based on the processing of event-related potentials after listening to words	Aachen Aphasia Test (AAT) consists of six description levels for spontaneous speech and five subtests: token test, repetition, written language, naming and comprehension. Snodgrass & Vanderwart (S&V) picture naming test (visual memory). Subtests of alertness, attention and flexibility of the standardized “Test of attentional performance” battery, digit span, Corsi span and semantic and phonological fluency.	Improvements in language/verbal skills, and reduced aphasia symptoms. AAT scores increased significantly after the intervention.
Kotov et al. [82]	Controlled, randomization and blinding not reported. No follow-up	*n* = 22 Age per group not reported. Total sample mean age = 61.1 ± 0.5 years	*n* = 22. The solely BCI-based intervention was regarded as the control, while the experimental group underwent BCI and multimodal stimulation		Kinesthetic feedback using an exoskeleton	A.R. Luria’s “10 Words” test (verbal memory, attention, and learning capacity), MoCA (cognitive screening), and Stroop test (inhibitory control)	Improvements in perception, construction, attention and memory. The experimental group had significant increases in MoCA visuospatial/executive and attention subscales. The control group had significant increases in the memory/delayed recall subscale. Mean increases in MoCA scores of 1.87 in the experimental and 1.19 in the control group.
Fateeva [80]	Controlled, randomized with a 1:1 ratio (random number generator), non-blinded (open-label). No follow-up	*n* = 14, mean age = 57.4 ± 6.9 years	*n* = 15, mean age = 57.0 ± 6.5 years Conventional cognitive rehabilitation		Visual feedback using task with a P300 speller	MoCA (cognitive screening), Schulte test (sustain attention), and A. R. Luria 10-word memorization test (verbal memory, attention, and learning capacity)	Improvements in attention and construction. Mean changes in MoCA scores of 2 in the BCI group and of 1.4 in the control group. Intergroup significant differences were only observed in the attention domain of MoCA.
Chen et al. [76]	Controlled, randomization and blinding not reported. Five follow-ups: 1, 2, 4 months, and 2, 4.5 years after intervention	*n* = 31 Age per group not reported. Total sample age range: 60 to 90 years	*n* = 69 35 patients received functional electrical stimulation, while 34 patients received conventional therapy		Kinesthetic and visual feedback using functional electrical stimulation controlled with hand motor intention	MoCA (cognitive screening)	Improvements in motor skills. Mean changes in MoCA scores of 1.6 ± 0.5 for the BCI group but were reverted to almost baseline after 4.5 years. MoCA scores decreased by −0.2 in the FES group, and increased in the conventional therapy group by 0.6 points.
Sebastian-Romagosa et al. [77]	Uncontrolled. Two follow-ups: 1 month and 6 months post intervention	*n* = 22, mean age = 53.8 ± 17.2 years	No		Kinesthetic and visual feedback using functional electrical stimulation controlled with hand or ankle motor imagery	MoCA (cognitive screening) and Color Stroop Test (inhibitory control)	Changes in specific cognitive domains were not detailed. MoCA changes were not significantly different after the intervention (median changes in MoCA scores of 0.5 [−0.5, 1.62]).
Zhao et al. [92]	Sham-controlled, randomized, double-blind. No follow-up	*n* = 14, mean age = 56.1 ± 11.5 years	*n* = 14, mean age = 50.1 ± 11.1 years Sham intervention with the BCI placed with feedback activation not related to intention		Kinesthetic and visual feedback using a lower limb robot	Loewenstein Occupational Therapy Cognitive Assessment (LOTCA), with five cognitive domains: orientation, visual and spatial perception, motor praxis, visuospatial function and cognitive organization	Changes in specific cognitive domains were not detailed, but the LOTCA measures sensation, motor skills and executive functioning. The increase in LOTCA scores was higher in the BCI group 14.9% compared to the sham group.
Liu et al. [81]	Controlled, randomized, single-blind (assessment blinding). No follow-up	*n* = 30, median age = 52.5 (45.0, 59.3) years. Received BCI plus conventional rehabilitation	*n* = 30, median age = 53.0 (38.5, 59.5) years Conventional rehabilitation		Visual and somatosensory feedback with a virtual avatar and hand functional electrical stimulation	Attention Network Test (ANT) with 3 components: alertness, orientation, and executive control. Schulte Grid Test (sustain attention) and Symbol Digit Modalities Test (SDMT) (attention and processing speed)	Improvements in attention. Significant lower response times in the Attention Network test scores of the BCI group of −44.5 ms, compared to the control group, 28 ms.
Isakova et al. [78]	Controlled, randomized (simple randomization), blinding not reported. No follow-up	*n* = 70, mean age for P300 group = 59.7 ± 12.3 years, mean age for MI group = 60.7 ± 9.2 years. Received BCI plus biofeedback	*n* = 19, mean age = 65.4 ± 8.0. Conventional therapy		37 patients received visual feedback based on P300 and 33 patients received somatosensory robotic feedback based on sensorimotor rhythms	MoCA scale (cognitive screening), subtest 9 of the Wechsler test (numeric reasoning), Kohs blocks (visuospatial function), Schulte tables (sustain attention), and A. R. Luria 10-word test (verbal memory, attention, and learning capacity)	Improvements in perception, orientation, executive functions and memory. Median changes in MoCA scores of 2 points in the BCI groups, while changes in MoCA scores were not significant in the control group.
Lim et al. [86]	Waitlist-controlled, randomized using stratification by gender and age with a 1:1 ratio (random permuted block sizes of 4 and 6), outcome assessor blinding. Follow-up 4 weeks post intervention	*n* = 72, mean age = 8.7 ± 1.4 years	*n* = 72, mean age = 8.6 ± 1.7 years. BCI-based therapy after initially being waitlist-controls	Attention-deficit/hyperactivity disorder	Visual feedback with the control of a virtual avatar within a computer game	ADHD rating scale (ADHD-RS) (rate the frequency of ADHD symptoms in everyday life) with 2 components: (1) inattentive and (2) hyperactive impulsive.	Improvements in attention. ADHD-RS score change implied a reduction of 3.3 ± 5 points in the experimental group, significantly higher than the reduction of 1.4 ± 3.94 points observed in the control group.
Lim et al. [87]	Controlled, randomized with a 1:1 ratio (permuted block technique), outcome assessor blinding. No follow-up	*n* = 10, mean age = 10.3 ± 2.0 years	*n* = 10, mean age = 9.5 ± 1.3 years BCI-based therapy within clinical environment		Visual feedback with the control of a virtual avatar within a computer game within a home setting	ADHD rating scale (ADHD-RS) (rate the frequency of ADHD symptoms in everyday life) with 2 components: (1) inattentive and (2) hyperactive impulsive. Children’s Global Assessment Scale (CGAS) and the Clinical Global Impressions Scale (CGI) (functionality in everyday life)	Improvements in attention. ADHD-RS score change implied a reduction of 3.2 ± 6.2 points in the experimental group, not significantly different to the reduction of 3.9 ± 5.08 points observed in the control group.
Turan et al. [88]	Controlled, non-randomized, blinding not reported. No follow-up	*n* = 132. 44 patients with BCI plus methylphenidate (mean age = 9.7 ± 2.4, 44 patients with BCI plus citicoline (mean age = 10.4 ± 2.5), 44 patients with BCI-only (mean age = 11.3 ± 2.8)	*n* = 42, mean age = 11.3 ± 3.1. Citicoline-only		Visual feedback with the control of a virtual avatar within a computer game	Computerized Conners Continuous Performance Test–Third Edition (CPT-3) (sustained attention, inhibitory control, and response consistency). SNAP-IV scale (inattention and hyperactivity). Sluggish Cognitive Tempo (SCT) scale (inattention, dreaminess, mental fogginess), and the Revised Child Anxiety and Depression Scale (RCADS)	Improvements in attention. Statistically significant changes were not observed between groups, but intergroup significant changes were observed for the BCI plus methylphenidate group 11.41 ± 16.5.

### 4.3. Feedback Types and BCI Control Paradigms

Most BCI interventions for cognitive rehabilitation have relied on visual feedback, with P300 spellers being particularly used, due to this type of BCI application being the first to be developed within the BCI community. Although originally created for communication purposes, P300 spellers have potential for cognitive rehabilitation due to being intuitive, depending on a reliable control paradigm, and being especially suitable for attention, executive functioning, and language/verbal skills, as implied by the few studies that have targeted aphasia. P300 speller feedback is depicted in Figure 4a. Furthermore, most of the ADHD patients that received a BCI-based intervention controlled a virtual avatar in a computer game, depicted in Figure 4b, which ran faster or slower depending on the classification of an attention state metric computed from frequency power features that first needed to be calibrated [86,93,94]. This type of computer gaming-based feedback seems to be promising not only for ADHD, but also for other pathologies/conditions, and has been reported to be accepted by elderly populations, which are more likely to have mild to severe cognitive impairment [95].

Kinesthetic feedback has also been reported across several of the assessed BCI studies, mainly involving robotic assistive devices or functional electrical stimulation, the latter depicted in Figure 4c [76]. Although this type of feedback is mainly controlled through movement-related paradigms, which are challenging to learn even for healthy populations, kinesthetic feedback has the advantage of complementing the cognitive domains that can be reinforced by BCIs with visual feedback, such as executive function, memory, perception, and construction.

Furthermore, BCIs that use a combination of motor control paradigms and visual feedback can also have positive effects for cognitive training. An example of this is the study of Gomez-Pilar et al. in which hand motor imagery was used to control complex visual feedback via two hand movement imagery tasks, as shown in Figure 4d [84]. Auditive feedback, although technically the simplest of the feedback types employed, is the least reported across the assessed studies, even if it has potential for treating aphasia symptoms, as implied in the study by Musso et al. [75]. Therefore, the choice of feedback and the control paradigm used to elicit feedback are the two most important technical features that need to be taken into account when designing a BCI for cognitive rehabilitation, since they will directly involve the cognitive domains that will be rehabilitated.

### 4.4. Technical Features of BCI Systems for Cognitive Rehabilitation

A BCI designed for cognitive rehabilitation needs to fulfill technical specifications that are also needed for other types of BCI applications. Technical features of the BCI systems used in the reviewed studies are shown in Table 4. Since all the assessed studies used EEG-based systems, it is important to acknowledge that state-of-the-art EEG acquisition systems for BCIs feature 16 to 24 bits of analog-to-digital conversion resolution, with most of them using delta–sigma technology [96,97]. This specification is crucial for achieving a high signal-to-noise ratio, which is essential for extracting features from EEG signals. Furthermore, the limited spatial resolution of EEG is also a challenge for discriminating between tasks ranging from a P300 evoked potential to sensorimotor rhythm detection, with this limited spatial resolution being tackled mainly with a higher number of channels. Therefore, some portable EEG acquisition devices commonly used for neurofeedback, mainly those that record one or two channels within the forehead with low analog-to-digital conversion resolution, may be unsuitable for BCI-based cognitive rehabilitation. Nonetheless, it is worth noting that some BCI systems have reported positive cognitive outcomes with as few as two channels. Unlike neurofeedback applications that only require the presentation of real-time feedback based on levels of psychophysiological measurable metrics, BCI-based cognitive rehabilitation needs to incorporate algorithms that allow the system to extract features and classify between two or more tasks within the range of a second or less. This was a common feature among assessed studies, with the common spatial pattern algorithm and linear discriminant analysis reported in some cases [86,87,88].

Importantly, there is no evidence of studies that have used invasive BCIs for cognitive rehabilitation for the assessed pathologies/conditions. Invasive acquisition modalities such as ECOG could allow patients with severe cognitive and motor impairments to control a BCI system, making BCI cognitive rehabilitation for AD and Parkinson’s disease patients feasible. Therefore, invasive BCIs remain a potentially promising field that has not yet been explored for cognitive training.

An advantage of BCI-based cognitive rehabilitation over other BCI rehabilitation applications, such as movement-related rehabilitation, is that the technical complexity of the feedback can be simple. This simplicity is possible because visual feedback allows for significant recovery of cognitive functions, while the system can also accommodate more complex feedback, such as those based on robotic assistive devices or functional electrical stimulation. This means that investing resources in complex hardware systems for the feedback stage is not a requirement for cognitive rehabilitation based on BCIs.

The effects of BCI-based cognitive training have been reported across the attention, perception, language/verbal skills, memory, motor skills, construction, and executive functioning domains of cognition. This implies that this type of therapy has the potential to improve several cognitive domains. This capability is likely driven by diverse neuroplasticity processes enhanced through different feedback and control paradigms, which can even be tailored to target a specific domain, such as attention in ADHD [86,87]. Attention and language/verbal skills were the two most reported enhanced cognitive domains across studies. Enhancement in these domains can be clinically relevant across the complete range of assessed pathologies/conditions and thus provides evidence of the clinical potential of BCIs. However, caution must be taken due to the limitations of study samples and the lack of interventions that only use BCIs for cognitive intervention, as several studies combined BCIs with other types of interventions. Therefore, this can prevent discerning the effects of standalone BCI interventions.

The cognitive domains of sensation and processing speed were not identified in any of the studies as having been enhanced after BCI-based interventions. It is unclear if this is because these cognition domains were not specifically assessed, or because therapies did not target them. Evidence regarding whether BCI devices can have effects on these two domains will also provide a relevant contribution to the current state of knowledge.

BCI-based interventions, even if not aimed specifically for cognitive training, such as in the study of Sebastián-Romagosa et al., can still have positive effects in enhancing cognition domains [77]. Therefore, participants of other studies comprising BCI-based interventions could have had improvements in cognition; however, these studies that evaluated other outcomes are a missed opportunity to better understand the potential of BCI-based cognitive rehabilitation. Consequently, including at least one neuropsychological assessment in future BCI clinical trials that measures attention or verbal/language skills as a secondary outcome could aid in speeding the pace at which BCI-based cognitive rehabilitation evidence is acquired.

### 4.5. Methodological Limitations of the Existing Literature

There are some methodological issues across the presented studies that also need to be addressed. For example, the small sample size in some studies may limit the statistical power and generalizability of the reported findings. In addition, some studies lacked control groups, and although most of the controlled studies used randomization for allocation, the procedures were not consistently reported. Similarly, blinding was not explicitly stated in many studies and the outcome heterogeneity across studies may limit the comparison of findings, which could complicate the assessment of possible outcome reporting bias. Lastly, concurrent therapies provided to patients alongside a BCI intervention could complicate the identification of the specific BCI effect. Therefore, even though the presented findings provide promising data supporting the integration of BCI-based interventions into cognitive rehabilitation across different pathologies, the results should be interpreted cautiously. Ultimately, large-scale, well-designed randomized controlled trials remain essential to definitively establish their clinical efficacy.

## 5. Conclusions and Future Directions

To date, the amount of clinical evidence of BCI-based cognitive rehabilitation is still scarce compared to other types of BCI rehabilitation, such as motor rehabilitation. For AD and Parkinson’s disease, this can be understood as stemming from the difficulty that dementia poses for patients to comply with the tasks that are required for BCI control. However, in the case of MCI and ADHD, the lack of sufficient evidence is more difficult to elucidate. It would be expected that a higher number of studies, representing the bulk of the evidence, would have assessed BCI effects on MCI alone, but only one controlled study could be identified. With MCI being a likely precursor of dementia, the clinical value of assessing BCI-based cognitive training in this condition seems to be justified. Furthermore, validated clinical assessments designed specifically for MCI, such as the MoCA, already exist, which makes the clinical assessment of cognitive domains in MCI more straightforward compared to other neurological conditions. Therefore, the main obstacle for more studies seems to be the technological complexity that BCI systems present, making their accessibility within clinical environments limited to date.

The main question that remains unanswered with the current evidence is “Why is it necessary to use a BCI for cognitive rehabilitation?”. It can be argued that more accessible technologies related to computerized rehabilitation or neurofeedback have also shown promise for cognitive training. This is perhaps the most important question that must be answered before BCIs can even be considered a complementary cognitive therapy. The pathway to answering this question involves increasing the number of clinical trials, as stated before, alongside the standardization of BCI technical features. In particular, future efforts must focus on harmonizing feedback and control paradigms, and most importantly, unifying the outcome measures of these studies. The MoCA seems to be a potential candidate for this purpose, but its application represents a challenge if qualified neuropsychology personnel are not available, and it cannot replace condition-specific and domain-specific assessments. Also, except for a BCI system used for ADHD across three studies, most reported clinical trials involve single studies that have yet to be reproduced by other research groups or have advanced their applications into scenarios closer to clinical applications. This lack of reproducibility and translational approaches has also reduced the amount of evidence for BCIs in cognitive rehabilitation.

Another question that is still unanswered regarding BCI cognitive rehabilitation is “What type of BCI should I use?”. However, a more important question regarding BCI technology applications that researchers targeting cognitive training should ask is “What cognitive domain should the BCI target for enhancement?”, since the current reported evidence, even if it is scarce, has provided insight into which type of feedback and control paradigm can enhance one or more cognitive skills. For example, for targeting language/verbal skills, P300 spellers have been shown to be a suitable approach in aiding aphasia stroke patients to rehabilitate these skills. In the case of targeting MCI, motor-related paradigms with different tasks and a variety of visual feedback, resembling a serious game application, can be used to target several cognitive domains. However, more information is needed regarding unassessed BCI control paradigms, such as SSVEPs, which could increase different cognitive domain skills compared to those enhanced with P300 or sensorimotor rhythms, since they remain unreported for cognitive training. Therefore, feasibility studies regarding the effects of unassessed paradigms and feedback types could make an important contribution to the current state of knowledge. This type of BCI design phase is the most important from both a clinical and engineering perspective and needs to be carefully thought out to make rehabilitation plausible without burdening patients with excessive mental workloads that could negatively impact cognitive rehabilitation outcomes.

Most of the reported clinical trials involved stroke patients; although evidence is still scarce compared to BCI-based motor rehabilitation, the limited number of studies suggest that BCIs have potential for stroke cognitive rehabilitation. This is important, especially for patients with severe motor disabilities, since BCIs aimed at cognition can provide an opportunity for these patients to rehabilitate with minimal motor outputs—such as with their gaze using a P300 speller—allowing them to reduce the degree of functional disability by targeting memory and language/verbal skills. In theory, and from the scarce reported evidence, BCI-based training seems to be specially suited for treating aphasia; however, its effects have been assessed in less than 30 patients, showcasing the need to perform additional studies. Since stroke is a pathology for which BCI-based motor rehabilitation has shown favorable clinical effects and with the constant reporting of clinical trials, future research protocols could take into account the measurement of at least one cognitive domain, such as attention or executive functioning. By doing this, future clinical trials will also provide information regarding BCI cognitive effects. Existing studies already report that kinesthetic feedback, such as functional electrical stimulation or robotic assistive devices, can enhance, to a certain extent, cognitive skills.

It is important to mention that all of the assessed studies have involved non-invasive BCI systems, particularly EEG-based BCIs. This is likely due to EEG’s good acceptability by users, easy setup due to the widespread use of EEG caps marked with the 10-10 international system positions, coupled to active electrodes in research-grade systems, and good time resolution within the millisecond range. Although EEG offers the above-mentioned advantages, it does present an important restriction, a low spatial resolution. Other non-invasive acquisition techniques such as fMRI or MEG could allow for a more exact control of BCI applications. However, their limited availability makes them difficult to apply within a clinical environment. Another acquisition technique is endovascular electrical recording, which offers high spatial and temporal resolution. It has been theorized that BCI systems comprising invasive acquisition techniques could increase the probability of patients with severe cognitive impairment voluntarily controlling deep brain stimulation as feedback, as reviewed by Sun et al. and Ognard et al. [56,98]. However, invasive acquisition techniques and deep brain stimulation, which do not constitute a closed-loop BCI by itself, present unresolved issues, such as risks due to surgical procedures. Furthermore, ethical concerns exist regarding the consent and enrollment of patients with severe cognitive impairment in experimental invasive procedures. In addition, some of these invasive acquisition modalities, including endovascular applications, still lack evidence in humans regarding their efficacy in cognitive rehabilitation.

Due to the complexity of setting up such BCIs and their inherent increased thrombosis risk in AD patients, an endovascular BCI designed for cognitive training has yet to be reported. Nonetheless, the feasibility of long-term recordings has been demonstrated in preclinical and clinical studies, highlighting their potential [56].

Finally, although current evidence is still insufficient to support the use of BCIs for cognitive rehabilitation outside of research scenarios in any of the assessed pathologies/conditions, the reported evidence supports positive effects of BCIs for enhancing cognitive skills. Furthermore, BCI technical features seem to be related to the types of cognitive domains that can be targeted, with MCI, stroke, and ADHD being the conditions for which higher evidence could be acquired in the short or medium term. With millions of people suffering from cognitive impairment due to the pathologies and conditions analyzed in the present review, the assessment of BCI-based interventions for cognitive rehabilitation is justified from both a clinical and ethical perspective. However, limited BCI technology accessibility and the scarcity of randomized controlled clinical trials remain the primary causes for the lack of evidence that plagues the potential application of BCIs in this field.

## Figures and Tables

**Figure 1:**
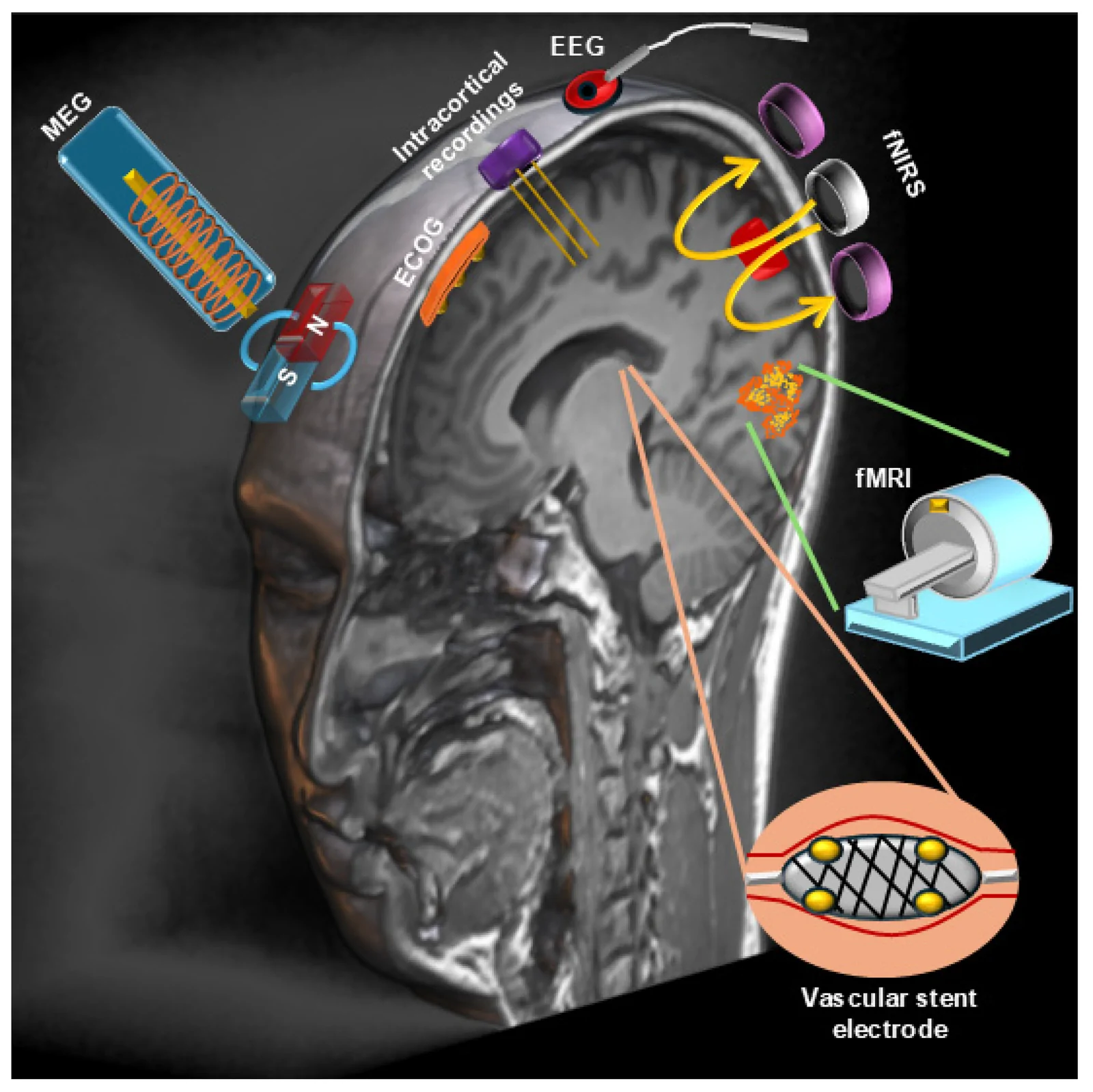
Signal acquisition modalities used in brain–computer interfaces. Electrocorticography (ECOG) and intracortical recordings require surgery for placing electrodes that have direct contact with the brain or vascular tissue. Electroencephalography (EEG) and functional near-infrared spectroscopy (fNIRS) require the placement of electrodes/optodes on the scalp but do not have direct contact with the brain. Magnetoencephalography (MEG) and functional magnetic resonance imaging (fMRI) do not require direct physical contact with the user.

**Figure 2:**
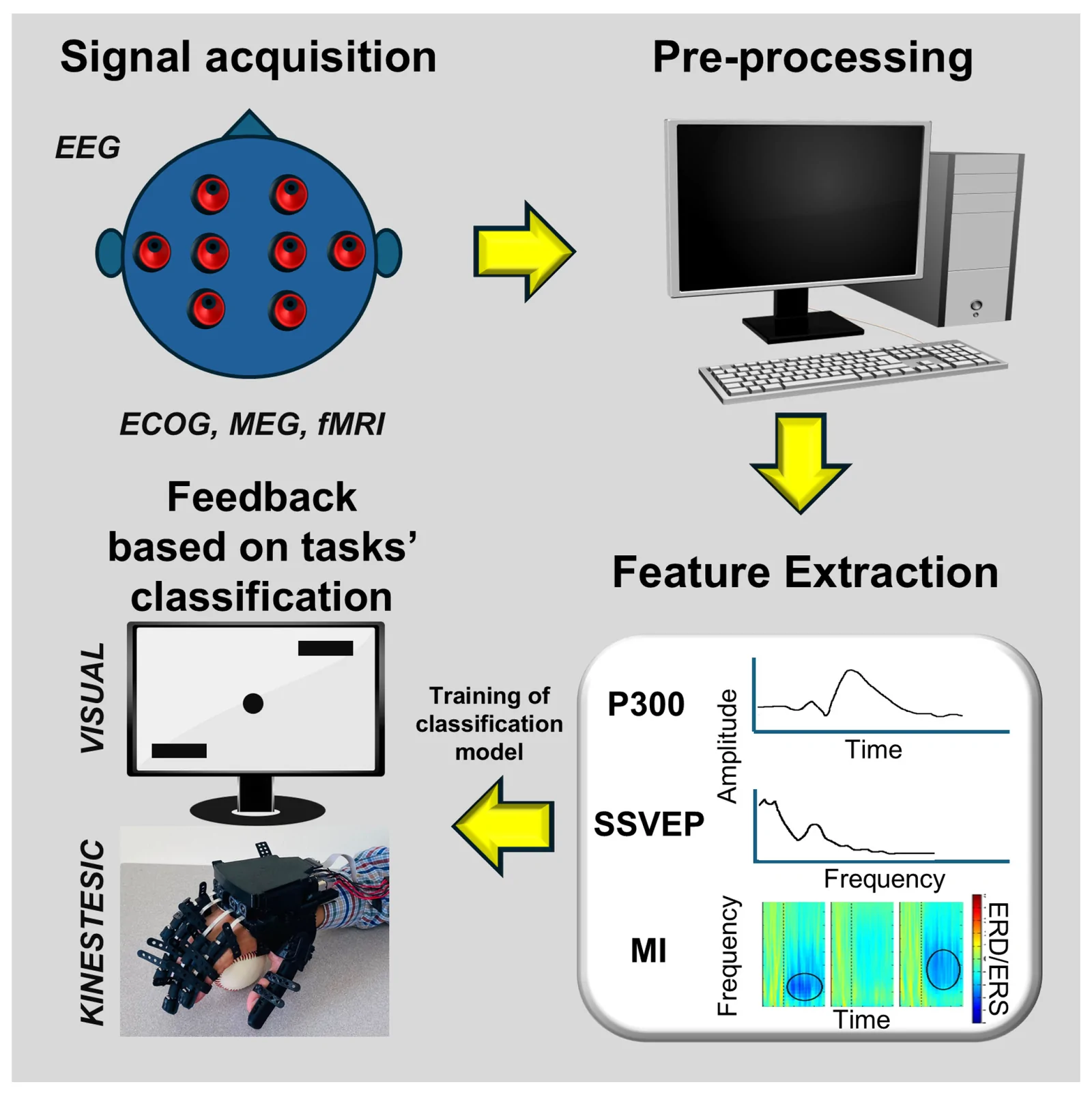
Stages of a brain–computer interface. Control paradigms are divided between those that extract features in the time domain, like the P300 potential (P300), in the frequency domain, such as Steady-State Visual Evoked Potentials (SSVEPs), or in the time–frequency domain, such as motor imagery or motor intention (MI). Event-related desynchronization/synchronization (ERD/ERS) can be computed in the time-frequency domain for assessing MI.

**Figure 3:**
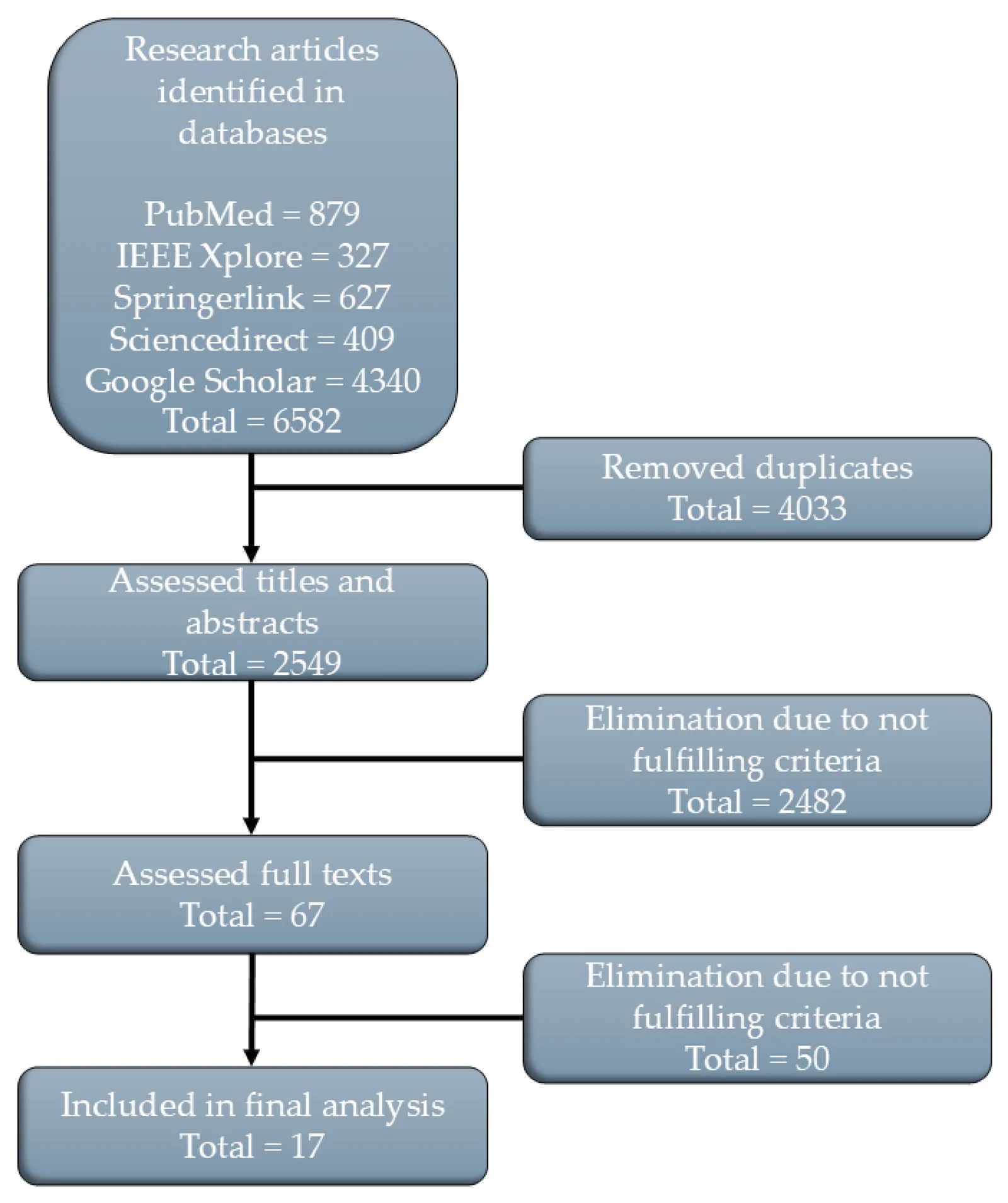
Detailed literature search and screening workflow.

**Figure 4:**
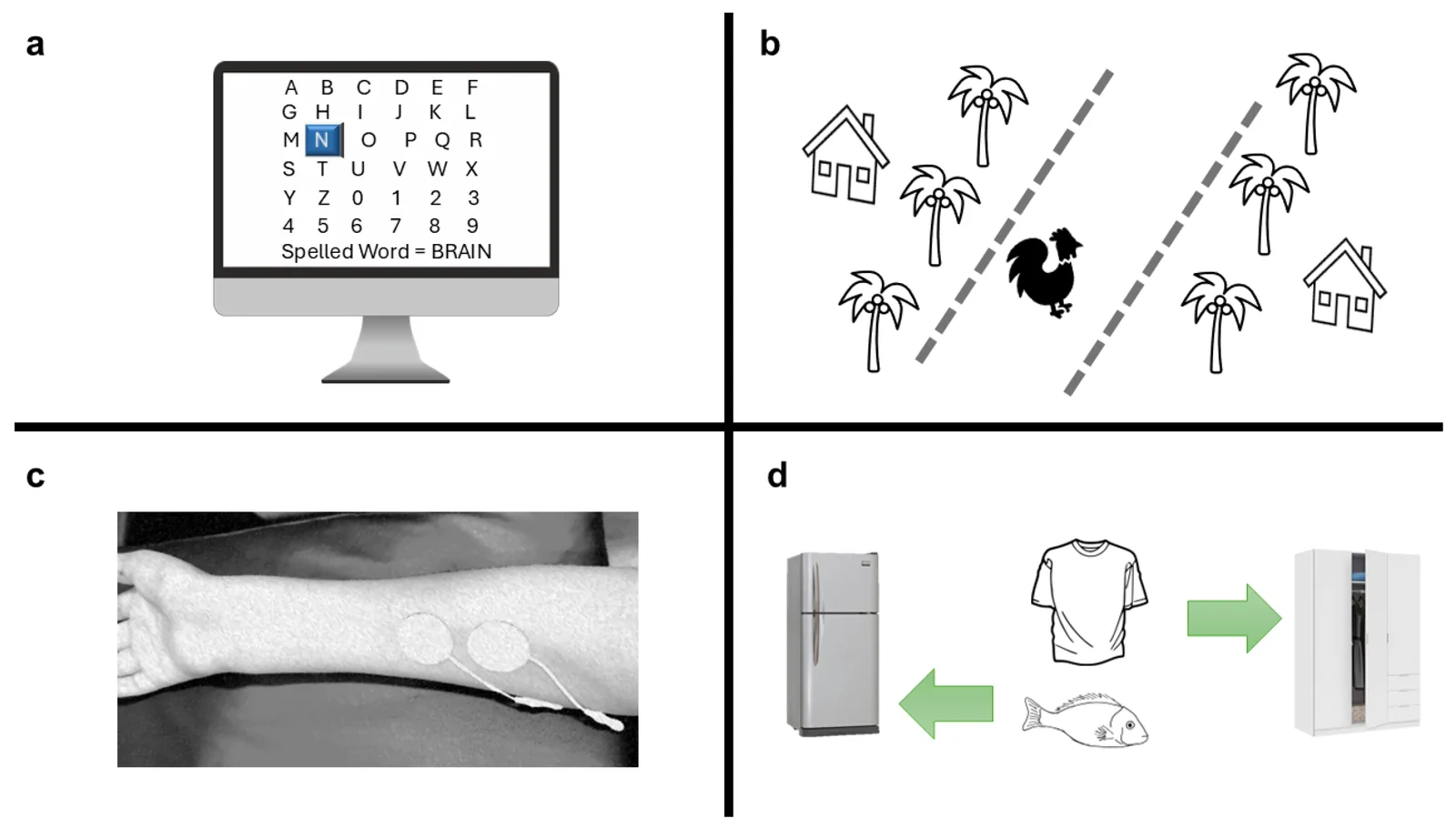
Feedback types used in studies that have contributed evidence of the cognition effects of brain–computer interface-based training. (**a**) P300 speller cognitive training for stroke aphasia [74]. (**b**) The control of an animal avatar within a virtual environment for attention-deficit/hyperactivity disorder [86]. (**c**) Functional electrical stimulation electrodes placed over the common flexor muscle for hand/wrist passive movement for stroke rehabilitation [76]. (**d**) The association of items with their storage places using right or left-hand motor imagery for cognitive training in the elderly [84].

**Table 1:** Treatment strategies approved by FDA for cognitive impairment [49,50,51,52].

Cognitive Disorder	Treatment	Mechanism of Action	Dose/Effects	FDA Year Approbation
Alzheimer disease	Donepezil	Reversible acetylcholinesterase (AChE) inhibitor	5–10 mg once-daily doses to be effective in reducing tau protein expression, decreasing hippocampal atrophy, lowering serum Aβ levels, and mildly improving cognitive performance.	1996
	Rivastigmine	dual inhibitor of AChE and butyrylcholinesterase	4.6 mg to 17.4 mg/day to be effective in delaying cognitive and behavior decline.	2000
	Galantamine	Reversible competitive acetylcholinesterase inhibitor	Improve function, cognition, and activities of daily living in a very short span of time (around 6 months) in patients with mild-to-moderate AD.	2001
	Memantine	Blocking current flow through channels of the glutamatergic NMDA receptors and inhibiting the influx of calcium into cells	10–20 mg/day Enhances synaptic plasticity in the brain, thereby improving memory and protecting against excitotoxicity.	2003
	Lecanemab	Anti-amyloid immunotherapy	Reduces amyloid plaques; approved for MCI and mild dementia with positive biomarkers.	2023
	Donanemab	Anti-amyloid immunotherapy	Reduces amyloid plaques; approved for MCI and mild dementia with positive biomarkers.	2024
Post-stroke cognitive impairment	Transcranial direct current stimulation (tDCS) and repetitive transcranial magnetic stimulation (rTMS)	Non-invasive brain stimulation	Statistically significant mean difference favoring brain stimulation therapies over control (sham) interventions but with high heterogeneity between studies.	There is no approved intervention for this indication to date.
ADHD	Metylphenydate Amphetamine-based	Stimulants of CNS (blocks the reuptake of the neurotransmitters norepinephrine (NE) and dopamine in presynaptic neurons)	10–60 mg/day Reduces hyperactivity and impulsivity and help children to concentrate.	1961
	Atomoxetine	Norepinephrine-Selective Reuptake Inhibitor	10–100 mg/day Improves focus, reduces impulsivity and provides emotional balance, with a lower risk of abuse or dependence.	2002
	Centanafadine	Norepinephrine, dopamine, and serotonin reuptake inhibitor	80–300 mg/day Lowers scores of inattention, hyperactivity, and impulsivity. Reduced risk of abuse, and best profile in patients with anxiety.	2026

**Table 2:** Specific database search syntaxis. Queries involving the ScienceDirect and Google Scholar searches were repeated two times. For this second search in ScienceDirect, the term “Cerebrovascular disease” was replaced with the term “stroke”. This was done due to restrictions on the number of permitted Boolean operators per search query. For Google Scholar, the term “Cerebrovascular disease” was replaced with the term “stroke”, and the term “Neurorehabilitation” was eliminated. This was performed to narrow the number of results per query due to the large number of studies that have assessed stroke neurorehabilitation. Duplicates were eliminated for the ScienceDirect and Google Scholar queries.

Dataset	Date of Search	Search Strings
PubMed	1 July 2026	(Brain Computer Interface OR BCI) AND (Cognitive Rehabilitation OR Neurorehabilitation) AND (Alzheimer’s Disease OR Parkinson’s OR Mild Cognitive Impairment OR MCI OR Cerebrovascular Disease OR stroke OR ADHD)
ScienceDirect	1 July 2026	(“Brain Computer Interface” OR BCI) AND (“Cognitive Rehabilitation” OR “Neurorehabilitation”) AND (“Alzheimer’s Disease” OR “Parkinson’s Disease” OR “Mild Cognitive Impairment” OR “Cerebrovascular disease” (alternately: stroke) OR ADHD)
Springerlink	1 July 2026	(“Brain Computer Interface” OR BCI) AND (“Cognitive Rehabilitation” OR “Neurorehabilitation”) AND (“Alzheimer’s Disease” OR “Parkinson’s Disease” OR “Mild Cognitive Impairment” OR “Cerebrovascular disease” OR stroke OR ADHD)
IEEE Xplore	1 July 2026	(Brain Computer Interface OR BCI) AND (Cognitive Rehabilitation OR Neurorehabilitation) AND (Alzheimer’s Disease OR Parkinson’s Disease OR Mild Cognitive Impairment OR Cerebrovascular disease OR stroke OR ADHD)
Google Scholar	1 July 2026	(“Brain Computer Interface” OR BCI) AND (“Cognitive Rehabilitation” OR neurorehabilitation) AND (“Alzheimer’s Disease” OR “Parkinson’s Disease” OR “Mild Cognitive Impairment” OR “Cerebrovascular disease” (alternately: stroke) OR ADHD)

**Table 4:** The technical features of brain–computer interfaces for cognitive rehabilitation used in the reviewed studies. The BCI acquisition modality for all studies was electroencephalography (EEG). NS = Not specified.

Study	EEG Channel Number/Resolution	Extracted Features	Condition	Control Paradigm	Classifier/Online Performance	Feedback Modality	Training Sessions	Session Duration	Adverse Events/Tolerability
Turconi et al. [72]	11/ NS	Sensorimotor rhythm event-related desynchronization	Parkinson’s Disease	Motor imagery	NS/NS	Visual	15	1.5–2 h, 2–3 times per week	NS
Gomez-Pilar et al. [84]	8/ 24-bit, active electrodes	Alpha and beta event-related desynchronization/synchronization	Old age > 65 years	Motor imagery	NS/NS	Visual	5	90 min	NS
Mendoza-Laiz [85]	16/ 24-bit, active electrodes	Alpha and beta event-related desynchronization/synchronization	Mild cognitive impairment	Motor imagery	NS/NS	Visual	5 NFT + 5 WMT	NFT ~60 min; WMT ~20 min	NS
Kleih et al. [74]	12/24-bit, passive electrodes	P300 maximum amplitude from 250 ms to 600 m	Stroke	Visual P300 speller (oddball paradigm)	Stepwise linear discriminant analysis/NS	Visual	1–6 B (A: 6; B: 3; C: 3; D: 3; E: 1)	First: 2 h (including TAP assessment); subsequent: 1.5 h.	NS
Tonin et al. [91]	17/ 24-bit	Fisher score computed from alpha and beta power		Covert visuospatial attention orienting	Quadratic discriminant analysis/55% to 60%	Visual	6 (first 2 calibration; subsequent in online modality)	NS	NS
Kleih et al. [73]	12/ 16-bit	P300 averaged amplitude from 250 ms to 600 ms		Visual P300 speller (oddball paradigm)	Linear discriminant analysis/87.9% to 59.7%	Visual	12–20 (20 planned)	1–1.5 h (including electrode preparation)	NS
Musso et al. [75]	31/16-bit, passive electrodes	P300 averaged amplitude from 250 ms to 600 m		Auditory target-word detection (modified oddball paradigm; AMUSE-based)	Regularized linear discriminant analysis/NS	Auditory and visual	Mean 14.9 (4 days/week; until 30 h effective training)	~60–150 min	Undesired finger motor activity in P2; training stopped after 24 h/tolerability: NS
Kotov et al. [82]	NS	Motor imagery-related features		Kinesthetic motor imagery	NS/NS	Visual, auditory and tactile	8–10	30 min, with 3–5 min breaks.	NS
Fateeva [80]	8/NS	P300-derived features		Visual P300 speller	NS/NS	Visual	10 (daily)	≤1 h	NS
Chen et al. [76]	24/NS	Common spatial patterns log-variance features; personalized EEG band in alpha or beta.		Motor imagery	Linear discriminant analysis/NS	Visual and functional electrical stimulation	24 (3/week for 8 weeks)	40 min	NS
Sebastian-Romagosa et al. [77]	16/NS	Common spatial pattern log-variance features; EEG band 8–30 Hz		Motor imagery	Linear discriminant analysis/82.68% ± 10.05).	Visual and functional electrical stimulation	25 (3/week)	~1 h (including preparation and cleaning)	No adverse events indicated/tolerability: NS
Zhao et al. [92]	6/ 24-bit, active electrodes	Steady-state motion visual evoked potentials		Steady-state motion visual evoked potential	NS/NS	Visual and robot-assisted movement	24 (6/week × 4 weeks)	30 min	No adverse events indicated/procedure indicated as well tolerated
Liu et al. [81]	14/ 24-bit passive electrodes	Features extracted during motor imagery of different parts of the upper extremity		Motor imagery	NS/NS	Visual, auditory and FES	15 (5/week × 3 weeks)	20 min	NS
Isakova et al. [78]	Neurochat NS Exohand-2 NS	P300 Mu rhythm-derived power features		P300 event related potentials Mu rhythm	NS/NS	BFB BFF	8–10	30 min 20–30 min	NS
Lim et al. [86]	2 (dry electrodes)/NS	Time-domain features computed from filter-bank common spatial patterns and an attention-level metric calculated from these features	Attention-deficit/hyperactivity disorder	Attention-based BCI	Support vector machine/NS	Visual	24 intensive sessions over 8 weeks + 3 maintenance sessions over the next 12 weeks	NS	11/172 (6.4%) reported ≥ 1 AE; headache most common followed by dizziness; none severe or requiring medical treatment/reported as safe, engaging and well tolerated
Lim et al. [87]	2 (dry electrodes)/NS	Time-domain features computed from filter-bank common spatial patterns and an attention-level metric calculated from these features		Attention-based BCI	Support vector machine/NS	Visual	24 (3/week × 8 weeks)	NS	Mild fatigue in one; increased pre-existing vocal tics on game days in one; neither severe nor required treatment/tolerability: NS
Turan et al. [88]	2/NS	Time-domain features computed from filter-bank common spatial patterns and an attention-level metric calculated from these features		Attention-based BCI	Support vector machine/NS	Attention-based BCI	24 (3/week × 8 weeks)	20 min of gameplay/session	Adverse events: NE/indicated as well tolerated

## Data Availability

No new data were created or analyzed in this study. Data sharing is not applicable to this article.

## Abbreviations

The following abbreviations are used in this manuscript:

BCI	Brain–computer interface
AD	Alzheimer’s disease
MCI	Mild cognitive impairment
MoCA	Montreal Cognitive Assessment
EEG	Electroencephalography
MEG	Magnetoencephalography
fNIRS	Functional near-infrared spectroscopy
fMRI	Functional magnetic resonance imaging
SSVEP	Steady-state visual evoked potential
MI	Motor imagery/intention
MMSE	Mini-Mental State Examination
TAP	Test battery for Attention Performance
AAT	Aachen Aphasia Test
LOTCA	Loewenstein Occupational Therapy Cognitive Assessment
ANT	Attention Network Test
ADHD	Attention-deficit/hyperactivity disorder
CPT-3	Conners Continuous Performance Test–Third Edition

## References

[1] Wong K.-H., Anderson C.D., Peterson C., Bouldin E., Littig L., Krothapalli N., Francis T., Kim Y., Cucufate G., Rosand J. (2025). Rising Cognitive Disability as a Public Health Concern Among US Adults. Neurology.

[2] Pais R., Ruano L., Carvalho P.O., Barros H. (2020). Global Cognitive Impairment Prevalence and Incidence in Community Dwelling Older Adults—A Systematic Review. Geriatrics.

[3] Salari N., Lotfi F., Abdolmaleki A., Heidarian P., Rasoulpoor S., Fazeli J., Najafi H., Mohammadi M. (2025). The Global Prevalence of Mild Cognitive Impairment in Geriatric Population with Emphasis on Influential Factors: A Systematic Review and Meta-Analysis. BMC Geriatr..

[4] Ge S., Zhu Z., Wu B., McConnell E.S. (2018). Technology-Based Cognitive Training and Rehabilitation Interventions for Individuals with Mild Cognitive Impairment: A Systematic Review. BMC Geriatr..

[5] Hu M., Wu X., Shu X., Hu H., Chen Q., Peng L., Feng H. (2021). Effects of Computerised Cognitive Training on Cognitive Impairment: A Meta-Analysis. J. Neurol..

[6] Kivipelto M., Mangialasche F., Snyder H.M., Allegri R., Andrieu S., Arai H., Baker L., Belleville S., Brodaty H., Brucki S.M. (2020). World-Wide FINGERS Network: A Global Approach to Risk Reduction and Prevention of Dementia. Alzheimer’s Dement..

[7] Martin S.S., Aday A.W., Allen N.B., Almarzooq Z.I., Anderson C.A.M., Arora P., Avery C.L., Baker-Smith C.M., Bansal N., Beaton A.Z. (2025). 2025 Heart Disease and Stroke Statistics: A Report of US and Global Data From the American Heart Association. Circulation.

[8] Giacobini M., Ahnemark E., Medin E., Freilich J., Andersson M., Ma Y., Ginsberg Y. (2023). Epidemiology, Treatment Patterns, Comorbidities, and Concomitant Medication in Patients with ADHD in Sweden: A Registry-Based Study (2018–2021). J. Atten. Disord..

[9] Harvey P.D. (2019). Domains of Cognition and Their Assessment. Dialogues Clin. Neurosci..

[10] Arvanitakis Z., Shah R.C., Bennett D.A. (2019). Diagnosis and Management of Dementia: Review. JAMA.

[11] Frisoni G.B., Hansson O., Nichols E., Garibotto V., Schindler S.E., van der Flier W.M., Jessen F., Villain N., Arenaza-Urquijo E.M., Crivelli L. (2025). New Landscape of the Diagnosis of Alzheimer’s Disease. Lancet.

[12] Frisoni G.B., Aho E., Brayne C., Ciccarelli O., Dubois B., Fox N.C., Frederiksen K.S., Gabay C., Garibotto V., Hofmarcher T. (2025). Alzheimer’s Disease Outlook: Controversies and Future Directions. Lancet.

[13] Walker Z., Possin K.L., Boeve B.F., Aarsland D. (2015). Lewy Body Dementias. Lancet.

[14] Carson N., Leach L., Murphy K.J. (2018). A Re-examination of Montreal Cognitive Assessment (MoCA) Cutoff Scores. Int. J. Geriatr. Psychiatry.

[15] Mitchell A.J., Shiri-Feshki M. (2009). Rate of Progression of Mild Cognitive Impairment to Dementia—Meta-analysis of 41 Robust Inception Cohort Studies. Acta Psychiatr. Scand..

[16] McGrattan A.M., Pakpahan E., Siervo M., Mohan D., Reidpath D.D., Prina M., Allotey P., Zhu Y., Shulin C., Yates J. (2022). Risk of Conversion from Mild Cognitive Impairment to Dementia in Low- and Middle-income Countries: A Systematic Review and Meta-analysis. Alzheimer’s Dement. Transl. Res. Clin. Interv..

[17] Chaudhary S., Kumaran S.S., Kaloiya G.S., Goyal V., Sagar R., Kalaivani M., Jaganathan N.R., Mehta N., Srivastava A. (2020). Domain Specific Cognitive Impairment in Parkinson’s Patients with Mild Cognitive Impairment. J. Clin. Neurosci..

[18] Overton M., Pihlsgård M., Elmståhl S. (2019). Diagnostic Stability of Mild Cognitive Impairment, and Predictors of Reversion to Normal Cognitive Functioning. Dement. Geriatr. Cogn. Disord..

[19] Sanz-Blasco R., Ruiz-Sánchez de León J.M., Ávila-Villanueva M., Valentí-Soler M., Gómez-Ramírez J., Fernández-Blázquez M.A. (2022). Transition from Mild Cognitive Impairment to Normal Cognition: Determining the Predictors of Reversion with Multi-state Markov Models. Alzheimer’s Dement..

[20] Hainsworth A.H., Markus H.S., Schneider J.A. (2024). Cerebral Small Vessel Disease, Hypertension, and Vascular Contributions to Cognitive Impairment and Dementia. Hypertension.

[21] Elendu C., Amaechi D.C., Elendu T.C., Ibhiedu J.O., Egbunu E.O., Ndam A.R., Ogala F., Ologunde T., Peterson J.C., Boluwatife A.I. (2023). Stroke and Cognitive Impairment: Understanding the Connection and Managing Symptoms. Ann. Med. Surg..

[22] El Husseini N., Katzan I.L., Rost N.S., Blake M.L., Byun E., Pendlebury S.T., Aparicio H.J., Marquine M.J., Gottesman R.F., Smith E.E. (2023). Cognitive Impairment After Ischemic and Hemorrhagic Stroke: A Scientific Statement From the American Heart Association/American Stroke Association. Stroke.

[23] Meng L., Zhao J., Liu J., Li S. (2019). Cerebral Small Vessel Disease and Cognitive Impairment. J. Neurorestoratology.

[24] Lahey B.B., Pelham W.E., Stein M.A., Loney J., Trapani C., Nugent K., Kipp H., Schmidt E., Lee S., Cale M. (1998). Validity of DSM-IV Attention-Deficit/Hyperactivity Disorder for Younger Children. J. Am. Acad. Child Adolesc. Psychiatry.

[25] Leffa D.T., Caye A., Rohde L.A. (2022). ADHD in Children and Adults: Diagnosis and Prognosis. New Discoveries in the Behavioral Neuroscience of Attention-Deficit Hyperactivity Disorder.

[26] Martin A.F., Rubin G.J., Rogers M.B., Wessely S., Greenberg N., Hall C.E., Pitt A., Logan P.E., Lucas R., Brooks S.K. (2025). The Changing Prevalence of ADHD? A Systematic Review. J. Affect. Disord..

[27] Grigore M., Ionică M.V., Pătru L., Gheorman V., Wagner A.P. (2025). Recent Advances in the Etiology and Neural Pathways Underlying Attention-Deficit and Hyperactivity Disorder. Curr. Health Sci. J..

[28] Folstein M.F., Folstein S.E., McHugh P.R. (1975). Mini-Mental State. J. Psychiatr. Res..

[29] Nasreddine Z.S., Phillips N.A., Bédirian V., Charbonneau S., Whitehead V., Collin I., Cummings J.L., Chertkow H. (2005). The Montreal Cognitive Assessment, MoCA: A Brief Screening Tool For Mild Cognitive Impairment. J. Am. Geriatr. Soc..

[30] Borson S., Scanlan J., Brush M., Vitaliano P., Dokmak A. (2000). The Mini-Cog: A Cognitive ?Vital Signs? Measure for Dementia Screening in Multi-Lingual Elderly. Int. J. Geriatr. Psychiatry.

[31] Larner A.J. (2020). Cognitive Screeners (2): Short Patient-Performance Scales (5–10 Min). Manual of Screeners for Dementia.

[32] Mitchell A.J. (2017). The Mini-Mental State Examination (MMSE): Update on Its Diagnostic Accuracy and Clinical Utility for Cognitive Disorders. Cognitive Screening Instruments.

[33] O’Connor D.W., Pollitt P.A., Hyde J.B., Fellows J.L., Miller N.D., Brook C.P.B., Reiss B.B. (1989). The Reliability and Validity of the Mini-Mental State in a British Community Survey. J. Psychiatr. Res..

[34] Carnero-Pardo C. (2014). ¿Es Hora de Jubilar al Mini-Mental?. Neurología.

[35] Tsoi K.K.F., Chan J.Y.C., Hirai H.W., Wong S.Y.S., Kwok T.C.Y. (2015). Cognitive Tests to Detect Dementia. JAMA Intern. Med..

[36] Davis D.H., Creavin S.T., Yip J.L., Noel-Storr A.H., Brayne C., Cullum S. (2021). Montreal Cognitive Assessment for the Detection of Dementia. Cochrane Database Syst. Rev..

[37] Larner A.J. (2020). Cognitive Screeners (3): Longer Patient-Performance Scales (>10 Min). Manual of Screeners for Dementia.

[38] Abayomi S.N., Sritharan P., Yan E., Saripella A., Alhamdah Y., Englesakis M., Tartaglia M.C., He D., Chung F. (2024). The Diagnostic Accuracy of the Mini-Cog Screening Tool for the Detection of Cognitive Impairment—A Systematic Review and Meta-Analysis. PLoS ONE.

[39] Staffaroni A.M., Tsoy E., Taylor J., Boxer A.L., Possin K.L. (2020). Digital Cognitive Assessments for Dementia: Digital Assessments May Enhance the Efficiency of Evaluations in Neurology and Other Clinics. Pract. Neurol. (Fort Wash. Pa.).

[40] Chib S., Sharma K., Singh R. (2025). Interventions of Cognitive Impairment in Older Adults: A Comprehensive Review. Aging Health Res..

[41] Masserini F., Baso G., Gendarini C., Pantoni L. (2023). Therapeutic Strategies in Vascular Cognitive Impairment: A Systematic Review of Population, Intervention, Comparators, and Outcomes. Alzheimer’s Dement..

[42] Sala G., Gobet F. (2017). Does Far Transfer Exist? Negative Evidence From Chess, Music, and Working Memory Training. Curr. Dir. Psychol. Sci..

[43] Chan A.T.C., Ip R.T.F., Tran J.Y.S., Chan J.Y.C., Tsoi K.K.F. (2024). Computerized Cognitive Training for Memory Functions in Mild Cognitive Impairment or Dementia: A Systematic Review and Meta-Analysis. npj Digit. Med..

[44] Horowitz S. (2006). Biofeedback Applications: A Survey of Clinical Research. Altern. Complement. Ther..

[45] Surmeli T., Eralp E., Mustafazade I., Kos H., Özer G.E., Surmeli O.H. (2016). Quantitative EEG Neurometric Analysis–Guided Neurofeedback Treatment in Dementia. Clin. EEG Neurosci..

[46] Lavy Y., Dwolatzky T., Kaplan Z., Guez J., Todder D. (2019). Neurofeedback Improves Memory and Peak Alpha Frequency in Individuals with Mild Cognitive Impairment. Appl. Psychophysiol. Biofeedback.

[47] Kober S.E., Schweiger D., Reichert J.L., Neuper C., Wood G. (2017). Upper Alpha Based Neurofeedback Training in Chronic Stroke: Brain Plasticity Processes and Cognitive Effects. Appl. Psychophysiol. Biofeedback.

[48] Luijmes R.E., Pouwels S., Boonman J. (2016). The Effectiveness of Neurofeedback on Cognitive Functioning in Patients with Alzheimer’s Disease: Preliminary Results. Neurophysiol. Clin./Clin. Neurophysiol..

[49] Mechler K., Banaschewski T., Hohmann S., Häge A. (2022). Evidence-Based Pharmacological Treatment Options for ADHD in Children and Adolescents. Pharmacol. Ther..

[50] Kreiger K., Weiss E., Fluri F. (2025). Novel Therapies for Post-Stroke Cognitive Impairment: A Systematic Review. Front. Neurol..

[51] Varadharajan A., Davis A.D., Ghosh A., Jagtap T., Xavier A., Menon A.J., Roy D., Gandhi S., Gregor T. (2023). Guidelines for Pharmacotherapy in Alzheimer’s Disease—A Primer on FDA-Approved Drugs. J. Neurosci. Rural Pract..

[52] Wu C.-K., Fuh J.-L. (2025). A 2025 Update on Treatment Strategies for the Alzheimer’s Disease Spectrum. J. Chin. Med. Assoc..

[53] Slutzky M.W., Vansteensel M.J., Herff C., Gaunt R.A. (2025). A Brain–Computer Interface Working Definition. Nat. Biomed. Eng..

[54] Graimann B., Allison B., Pfurtscheller G. (2009). Brain–Computer Interfaces: A Gentle Introduction. Brain-Computer Interfaces: Revolutionizing Human-Computer Interaction.

[55] He B., Yuan H., Meng J., Gao S. (2020). Brain–Computer Interfaces. Neural Engineering.

[56] Sun Y., Chen D., Ye Q., You Z., Zhao Z., Shi J., Sun H., Li S., Xu X., Xu Y. (2025). Applications of Endovascular Brain–Computer Interface in Patients with Alzheimer’s Disease. Research.

[57] Mellinger J., Schalk G., Braun C., Preissl H., Rosenstiel W., Birbaumer N., Kübler A. (2007). An MEG-Based Brain-Computer Interface (BCI). Neuroimage.

[58] Rahman M.A., Siddik A.B., Ghosh T.K., Khanam F., Ahmad M. (2020). A Narrative Review on Clinical Applications of FNIRS. J. Digit. Imaging.

[59] Glover G.H. (2011). Overview of Functional Magnetic Resonance Imaging. Neurosurg. Clin. N. Am..

[60] Polich J. (2007). Updating P300: An Integrative Theory of P3a and P3b. Clin. Neurophysiol..

[61] Norcia A.M., Appelbaum L.G., Ales J.M., Cottereau B.R., Rossion B. (2015). The Steady-State Visual Evoked Potential in Vision Research: A Review. J. Vis..

[62] Carrillo-de-la-Peña M.T., Galdo-Álvarez S., Lastra-Barreira C. (2008). Equivalent Is Not Equal: Primary Motor Cortex (MI) Activation during Motor Imagery and Execution of Sequential Movements. Brain Res..

[63] Arvaneh M., Guan C., Ang K.K., Ward T.E., Chua K.S.G., Kuah C.W.K., Ephraim Joseph G.J., Phua K.S., Wang C. (2017). Facilitating Motor Imagery-Based Brain–Computer Interface for Stroke Patients Using Passive Movement. Neural Comput. Appl..

[64] Pfurtscheller G., Lopes Da Silva F.H. (1999). Event-Related EEG/MEG Synchronization and Desynchronization: Basic Principles. Clin. Neurophysiol..

[65] Lotte F., Congedo M., Lécuyer A., Lamarche F., Arnaldi B. (2007). A Review of Classification Algorithms for EEG-Based Brain–Computer Interfaces. J. Neural Eng..

[66] Kumar R., Waisberg E., Ong J., Lee A.G. (2025). The Potential Power of Neuralink—How Brain-Machine Interfaces Can Revolutionize Medicine. Expert Rev. Med. Devices.

[67] Simeral J.D., Hosman T., Saab J., Flesher S.N., Vilela M., Franco B., Kelemen J.N., Brandman D.M., Ciancibello J.G., Rezaii P.G. (2021). Home Use of a Percutaneous Wireless Intracortical Brain-Computer Interface by Individuals With Tetraplegia. IEEE Trans. Biomed. Eng..

[68] Kachhadia M.P., Sibhai I., Vaghela R., Topiwala U., Shaikh J.D., Tsai M., Borad N., Simon M., Ilyas H., Zahra T. (2025). Open-Loop and Closed-Loop Neuromodulation Across Neurological Disorders Toward Personalized Brain Stimulation: A Narrative Review. Cureus.

[69] Baethge C., Goldbeck-Wood S., Mertens S. (2019). SANRA—A Scale for the Quality Assessment of Narrative Review Articles. Res. Integr. Peer Rev..

[70] Oh S.-J., Ryu J.-N. (2018). The Effect of Brain-Computer Interface-Based Cognitive Training in Patients with Dementia. J. Korean Soc. Phys. Med..

[71] Galvin-McLaughlin D., Klee D., Memmott T., Peters B., Wiedrick J., Fried-Oken M., Oken B., Erdogmus D., Smith D., Bedrick S. (2022). Methodology and Preliminary Data on Feasibility of a Neurofeedback Protocol to Improve Visual Attention to Letters in Mild Alzheimer’s Disease. Contemp. Clin. Trials Commun..

[72] Turconi M.M., Mezzarobba S., Franco G., Busan P., Fornasa E., Jarmolowska J., Accardo A., Battaglini P.P. BCI-Based Neuro-Rehabilitation Treatment for Parkinson’s Disease: Cases Report. Proceedings of the Trieste Symposium on Perception and Cognition (TSPC2014).

[73] Kleih S.C., Botrel L. (2024). Post-Stroke Aphasia Rehabilitation Using an Adapted Visual P300 Brain-Computer Interface Training: Improvement over Time, but Specificity Remains Undetermined. Front. Hum. Neurosci..

[74] Kleih S.C., Gottschalt L., Teichlein E., Weilbach F.X. (2016). Toward a P300 Based Brain-Computer Interface for Aphasia Rehabilitation after Stroke: Presentation of Theoretical Considerations and a Pilot Feasibility Study. Front. Hum. Neurosci..

[75] Musso M., Hübner D., Schwarzkopf S., Bernodusson M., LeVan P., Weiller C., Tangermann M. (2022). Aphasia Recovery by Language Training Using a Brain–Computer Interface: A Proof-of-Concept Study. Brain Commun..

[76] Chen S., Xie N., Tang Y., Ji Y., He Z., Wang Y., Huang X., Fu J., Ge M., Liu Q. (2025). Long-Term Brain–Computer Interface Functional Electrical Stimulation Enhances Neuroplasticity and Functional Recovery in Elderly Stroke: A 4.5-Year Longitudinal Study Integrating Electroencephalography Biomarkers and Clinical Assessments. Research.

[77] Sebastián-Romagosa M., Cho W., Ortner R., Sieghartsleitner S., Von Oertzen T.J., Kamada K., Laureys S., Allison B.Z., Guger C. (2023). Brain–Computer Interface Treatment for Gait Rehabilitation in Stroke Patients. Front. Neurosci..

[78] Isakova E.V., Kotov S.V., Borisova V.A. (2026). Effectiveness of Brain–Computer Interfaces with Biofeedback in the Rehabilitation of Cognitive Impairment after Stroke. Neurosci. Behav. Physiol..

[79] Lindvall E., Abzhandadze T., Quinn T.J., Sunnerhagen K.S., Lundström E. (2024). Is the Difference Real, Is the Difference Relevant: The Minimal Detectable and Clinically Important Changes in the Montreal Cognitive Assessment. Cereb. Circ. Cogn. Behav..

[80] Fateeva V.V., Kushnir A.B., Grechko A.V., Mayorova L.A. (2024). Rehabilitation of Patients with Post-Stroke Cognitive Impairments Using a P300-Based Brain–Computer Interface: Results of a Randomized Controlled Trial. Neurosci. Behav. Physiol..

[81] Liu X., Zhang W., Li W., Zhang S., Lv P., Yin Y. (2023). Effects of Motor Imagery Based Brain-Computer Interface on Upper Limb Function and Attention in Stroke Patients with Hemiplegia: A Randomized Controlled Trial. BMC Neurol..

[82] Kotov S.V., Isakova E.V., Slyun’kova E.V. (2019). Usage of Brain–Computer Interface+exoskeleton Technology as a Part of Complex Multimodal Stimulation in the Rehabilitation of Patients with Stroke. Zhurnal Nevrol. Psikhiatrii im. S.S. Korsakova.

[83] Li D., Li R., Song Y., Qin W., Sun G., Liu Y., Bao Y., Liu L., Jin L. (2025). Effects of Brain-Computer Interface Based Training on Post-Stroke Upper-Limb Rehabilitation: A Meta-Analysis. J. Neuroeng. Rehabil..

[84] Gomez-Pilar J., Corralejo R., Nicolas-Alonso L.F., Álvarez D., Hornero R. (2016). Neurofeedback Training with a Motor Imagery-Based BCI: Neurocognitive Improvements and EEG Changes in the Elderly. Med. Biol. Eng. Comput..

[85] Mendoza Laiz N., Del Valle Díaz S., Rioja Collado N., Gomez-Pilar J., Hornero R. (2018). Potential Benefits of a Cognitive Training Program in Mild Cognitive Impairment (MCI). Restor. Neurol. Neurosci..

[86] Lim C.G., Poh X.W.W., Fung S.S.D., Guan C., Bautista D., Cheung Y.B., Zhang H., Yeo S.N., Krishnan R., Lee T.S. (2019). A Randomized Controlled Trial of a Brain-Computer Interface Based Attention Training Program for ADHD. PLoS ONE.

[87] Lim C.G., Soh C.P., Lim S.S.Y., Fung D.S.S., Guan C., Lee T.-S. (2023). Home-Based Brain–Computer Interface Attention Training Program for Attention Deficit Hyperactivity Disorder: A Feasibility Trial. Child Adolesc. Psychiatry Ment. Health.

[88] Turan S., Çıray R.O. (2026). Comparative Effects of BCI-Based Attention Training, Methylphenidate, and Citicoline on Attention and Executive Function in School-Age Children: A Quasi-Experimental Study. Medicina.

[89] Wang B.-R., Zheng H.-F., Xu C., Sun Y., Zhang Y.-D., Shi J.-Q. (2019). Comparative Diagnostic Accuracy of ACE-III and MoCA for Detecting Mild Cognitive Impairment. Neuropsychiatr. Dis. Treat..

[90] Zarrella G.V., Kay C.D., Gettens K., Sherman J.C., Colvin M.K. (2023). Addenbrooke’s Cognitive Examination–Third Edition Predicts Neuropsychological Test Performance. J. Neuropsychiatry Clin. Neurosci..

[91] Tonin L., Pitteri M., Leeb R., Zhang H., Menegatti E., Piccione F., Millán J.d.R. (2017). Behavioral and Cortical Effects during Attention Driven Brain-Computer Interface Operations in Spatial Neglect: A Feasibility Case Study. Front. Hum. Neurosci..

[92] Zhao C.-G., Ju F., Sun W., Jiang S., Xi X., Wang H., Sun X.-L., Li M., Xie J., Zhang K. (2022). Effects of Training with a Brain–Computer Interface-Controlled Robot on Rehabilitation Outcome in Patients with Subacute Stroke: A Randomized Controlled Trial. Neurol. Ther..

[93] Qian X., Loo B.R.Y., Castellanos F.X., Liu S., Koh H.L., Poh X.W.W., Krishnan R., Fung D., Chee M.W., Guan C. (2018). Brain-Computer-Interface-Based Intervention Re-Normalizes Brain Functional Network Topology in Children with Attention Deficit/Hyperactivity Disorder. Transl. Psychiatry.

[94] Lim C.G., Lee T.S., Guan C., Fung D.S.S., Zhao Y., Teng S.S.W., Zhang H., Krishnan K.R.R. (2012). A Brain-Computer Interface Based Attention Training Program for Treating Attention Deficit Hyperactivity Disorder. PLoS ONE.

[95] Goumopoulos C., Drakakis E., Gklavakis D. (2023). Feasibility and Acceptance of Augmented and Virtual Reality Exergames to Train Motor and Cognitive Skills of Elderly. Computers.

[96] Lin C.-T., Wang Y., Chen S.-F., Huang K.-C., Liao L.-D. (2023). Design and Verification of a Wearable Wireless 64-Channel High-Resolution EEG Acquisition System with Wi-Fi Transmission. Med. Biol. Eng. Comput..

[97] Dou C., Luo J., Lv J., Wang D., Zhao B., Zou L. (2026). A Wireless EEG Acquisition System Capable of Real-Time BCI Applications. IEEE Sens. J..

[98] Ognard J., El Hajj G., Verma O., Ghozy S., Kadirvel R., Kallmes D.F., Brinjikji W. (2025). Advances in Endovascular Brain Computer Interface: Systematic Review and Future Implications. J. Neurosci. Methods.

